# Type I interferon shapes the quantity and quality of the anti‐Zika virus antibody response

**DOI:** 10.1002/cti2.1126

**Published:** 2020-04-26

**Authors:** Cheryl Yi‐Pin Lee, Guillaume Carissimo, Zheyuan Chen, Fok‐Moon Lum, Farhana Abu Bakar, Ravisankar Rajarethinam, Teck‐Hui Teo, Anthony Torres‐Ruesta, Laurent Renia, Lisa FP Ng

**Affiliations:** ^1^ Singapore Immunology Network Agency for Science, Technology and Research (A*STAR) Singapore; ^2^ NUS Graduate School for Integrative Sciences and Engineering National University of Singapore Singapore; ^3^ School of Medicine Dentistry & Biomedical Sciences Queen's University Belfast Belfast UK; ^4^ School of Biological Sciences Nanyang Technological University Singapore Singapore; ^5^ Institute of Molecular and Cell Biology Agency of Science, Technology and Research (A*STAR) Singapore; ^6^ Department of Biochemistry Yong Loo Lin School of Medicine National University of Singapore Singapore; ^7^ Institute of Infection and Global Health University of Liverpool Liverpool UK; ^8^ Present address: Institut Pasteur Unite de Pathogenie Microbienne Moleculaire Paris France

**Keywords:** antibodies, humoral response, mouse models, type I interferon, Zika virus

## Abstract

**Objectives:**

Zika virus (ZIKV) is a mosquito‐borne *flavivirus* that re‐emerged in 2015. The association between ZIKV and neurological complications initiated the development of relevant animal models to understand the mechanisms underlying ZIKV‐induced pathologies. Transient inhibition of the type I interferon (IFN) pathway through the use of an IFNAR1‐blocking antibody, MAR1‐5A3, could efficiently permit active virus replication in immunocompetent animals. Type I IFN signalling is involved in the regulation of humoral responses, and thus, it is crucial to investigate the potential effects of type I IFN blockade towards B‐cell responses.

**Methods:**

In this study, comparative analysis was conducted using serum samples collected from ZIKV‐infected wild‐type (WT) animals either administered with or without MAR1‐5A3.

**Results:**

Serological assays revealed a more robust ZIKV‐specific IgG response and subtype switching upon inhibition of type I IFN due to the abundance of antigen availability. This observation was corroborated by an increase in germinal centres, plasma cells and germinal centre B cells. Interestingly, although both groups of animals recognised different B‐cell linear epitopes in the E and NS1 regions, there was no difference in neutralising capacity. Further characterisation of these epitopes in the E protein revealed a detrimental role of antibodies that were generated in the absence of type I IFN.

**Conclusion:**

This study highlights the role of type I IFN in shaping the anti‐ZIKV antibody response to generate beneficial antibodies and will help guide development of better vaccine candidates triggering efficient neutralising antibodies and avoiding detrimental ones.

## Graphical abstract

Type I IFN signalling is involved in the regulation of humoral responses, and thus, it is crucial to investigate the potential effects of type I IFN blockade towards ZIKV‐induced B‐cell responses. In this study, comparative analysis was conducted using serum samples collected from ZIKV‐infected wild‐type (WT) animals either administered with or without MAR1‐5A3. Results showed animals that have their type I IFN response transiently suppressed displayed a more robust ZIKV‐specific IgG response and subtype switching, which was corroborated by a higher number of germinal centres in the spleen. In addition, several linear B‐cell epitopes were identified from the envelope and non‐structural 1 proteins; however, interestingly, the dominant regions recognised between both groups of animals are different. Further characterisation of these dominant epitopes revealed a detrimental role of antibodies that were generated in the absence of type I IFN.

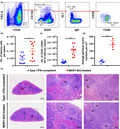

## Introduction

Zika virus (ZIKV) is an arbovirus belonging to the Flaviviridae family of the genus *Flavivirus*.^1^ In addition to transmission through mosquito vectors of the *Aedes* species, ZIKV can also be transmitted sexually, vertically from mother to child, and possibly through contact with body fluids.^2^, ^3^, ^4^ Since the 2015 epidemic in the Americas, ZIKV has garnered global attention because of its causal association with neurological complications, including Guillain–Barré syndrome and congenital ZIKV syndrome.^5^ Development of biological systems to understand the immunopathology and immune responses associated during ZIKV infection continues to draw interests.

Wild‐type (WT) animals are largely asymptomatic during ZIKV infection because of a robust innate immune response, rendering them unsuitable for study of disease‐related pathologies.^6^, ^7^ Since type I interferon (IFN) was shown to control viral replication and dissemination of several *flaviviruses*,^8^, ^9^ mouse models deficient in the type I IFN pathway, including IFNAR knock‐out (KO) and AG129, were developed for ZIKV studies.^7^, ^10^ These animals showed increased susceptibility to ZIKV infection with persisting viraemia and severe disease phenotypes.^7^, ^10^ However, the lethality and immuno‐deficient nature of these animals limit long‐term assessment of the host immune response during ZIKV infection.

To circumvent these limitations, a susceptible WT model was developed using the MAR1‐5A3 monoclonal antibody that transiently inhibits type I IFN receptor 1 (IFNAR1) at the time of infection.^11^ Upon treatment, these WT animals could support active ZIKV replication and recapitulate ZIKV pathologies depending on the administered dosage of MAR1‐5A3.^7^ Since ZIKV actively suppresses human type I IFN response,^12^, ^13^ it is hypothesised that MAR1‐5A3 treatment mimics this process during ZIKV infection in WT mice, thereby placing a high clinical value on this transient suppression of type I IFN model.

Earlier reports have shown that type I IFN is involved in the regulation of humoral response, either by upregulating antibody production,^14^, ^15^ or negatively impacting B‐cell responses.^16^, ^17^ Therefore, it is essential to understand the implications of type I IFN suppression on anti‐ZIKV humoral response in order to better understand and interpret results of preliminary vaccine studies using this model. In this study, we showed that the MAR1‐5A3‐treated WT mouse model presents alterations of ZIKV‐induced antibody response in both quantity and quality. Importantly, type I IFN suppression influenced the identified dominant B‐cell linear epitopes that stimulate an enhanced ZIKV infection. These findings highlight the importance to accurately interpret human serological data in order to guide the generation of stronger targeted B‐cell responses and avoid detrimental ones.

## Results

### Type I IFN suppression induces a more robust IgG response

**Figure 1:**
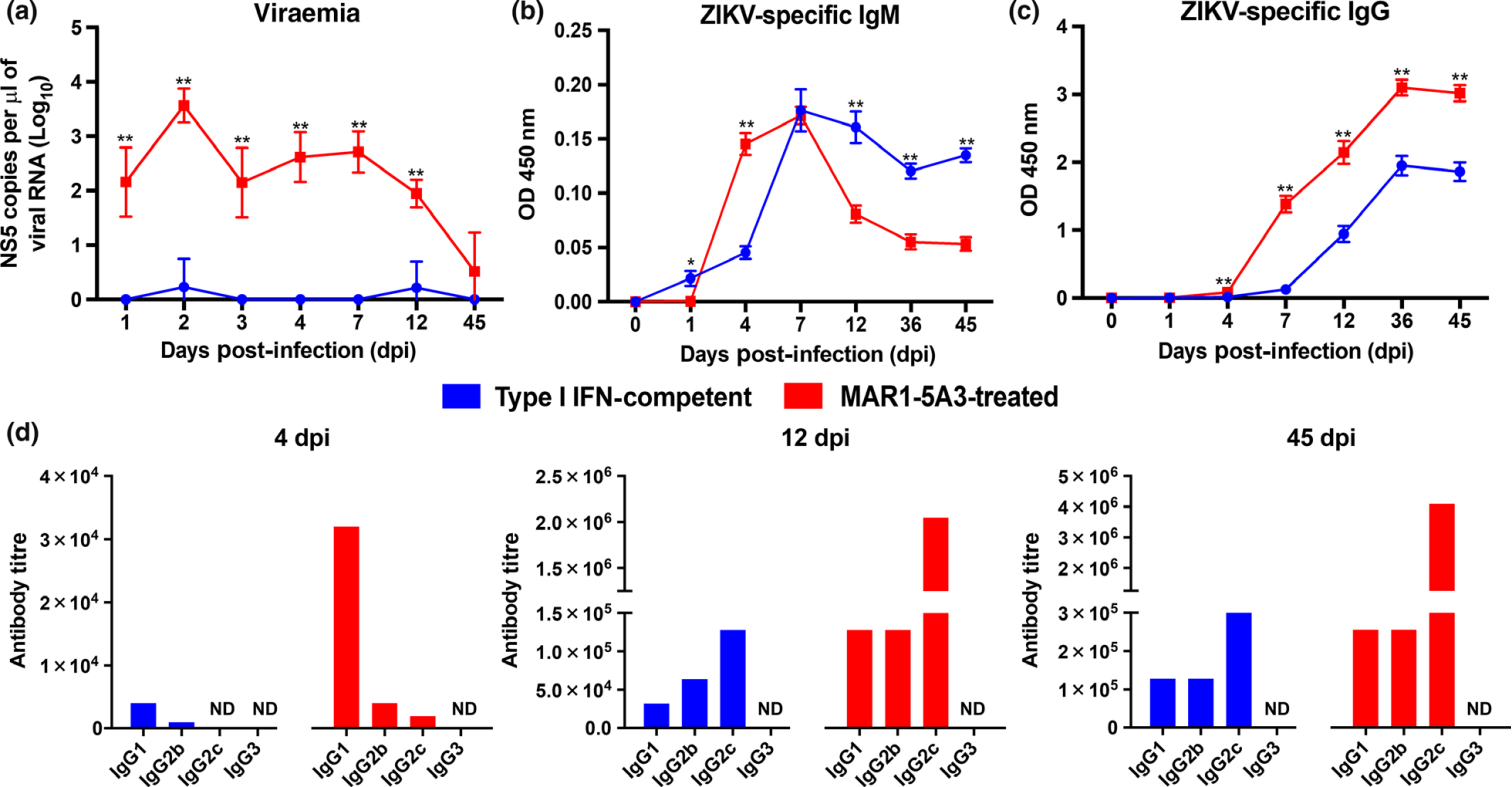
Type I IFN suppression supports virus replication and promotes a robust IgG antibody response. **(a)** Viraemia of ZIKV‐infected type I IFN‐competent (*n* = 5) and MAR1‐5A3‐treated (*n* = 5) WT mice. Mice were inoculated with 1 × 10^6^ PFU ZIKV i.v. by the retro‐orbital route. Two mg of MAR1‐5A3 was administered i.p. on the same day as infection. Data shown are expressed as mean ± SD. Statistical significance was measured using Mann–Whitney *U*‐test between type I IFN‐competent and MAR1‐5A3‐treated WT mice of the same days post‐infection (**P < 0.01). ZIKV virion‐based ELISA was conducted using pooled serum of *n* = 5 animals from **(b)** type I IFN‐competent and **(c)** MAR1‐5A3‐treated WT mice at 1:100 for IgM (top panel) and at 1:500 for IgG (bottom panel) detections. Data are represented as mean ± SEM of three independent experiments with five animals per group per experiment performed in two technical duplicates. **(d)** Levels of ZIKV‐specific IgG1, IgG2b, IgG2c and IgG3 were assessed by purified ZIKV virion‐based ELISA using pooled serum from *n* = 5 mice of respective groups collected at 4, 12 and 45 dpi. ZIKV‐specific IgG subtypes are expressed as antibody titre, defined as the greatest reacting dilution before the OD value reaches baseline control (pooled 0 dpi sera). ND, not detectable.

To assess differences in antibody profiles elicited between type I IFN‐competent and MAR1‐5A3‐treated mice, animals were inoculated through the retro‐orbital route with ZIKV. Administration of MAR1‐5A3 was done intraperitoneally to the latter group on the same day as infection. MAR1‐5A3‐treated animals showed high levels of viral RNA detectable in the circulation at 1 day post‐infection (dpi), with viraemia peaking at 2 dpi and no detectable viraemia by 45 dpi (Figure 1a). As expected, type I IFN‐competent WT mice did not sustain an active viral replication, with low to undetectable levels of ZIKV NS5 viral copies, corroborating previous studies (Figure 1a).^6^, ^7^, ^10^

To determine whether the difference in viraemia levels correlated with anti‐ZIKV humoral response levels, ZIKV‐specific antibody profiling was performed by virion‐based ELISA.^18^, ^19^ Prior to testing, the use of single and pooled mouse sera was compared, and results showed a similar response between both (Supplementary figure 1). As the amount of serum obtainable from each mouse is limited because of ethical concerns stated in our IACUC licence, subsequent experiments were performed using pooled mouse sera, which is also done by other studies.^20^, ^21^, ^22^ Both IgM and IgG were tested using pooled sera from five mice per group at 1, 4, 7, 12, 36 and 45 dpi (Figure 1b and c). ZIKV‐specific IgM was detected as early as 1 dpi, and production peaked at 7 dpi (Figure 1b and c). Interestingly, while the IgM response in MAR1‐5A3‐treated mice began to decline at 45 dpi, a sustainable level of IgM was observed in the type I IFN‐competent group, consistent with human observation.^23^ ZIKV‐specific IgG was detected at 4 dpi, and their levels increased substantially with time and remained constant even after viraemia was undetectable (Figure 1b and c). Notably, animals receiving MAR1‐5A3 treatment had a higher level of ZIKV‐specific IgG across all time points tested.

The effect of type I IFN suppression on IgG class switching during anti‐ZIKV response was then addressed. Subtyping of virus‐specific antibodies from pooled sera collected at 4, 12 and 45 dpi showed different dominant IgG subtypes at different time points, indicating efficient class switching from IgG1 to IgG2c for both groups of animals. IgG1 was dominant at 4 dpi, and IgG2c was the major subtype at 12 and 45 dpi (Figure 1d).

**Figure 2:**
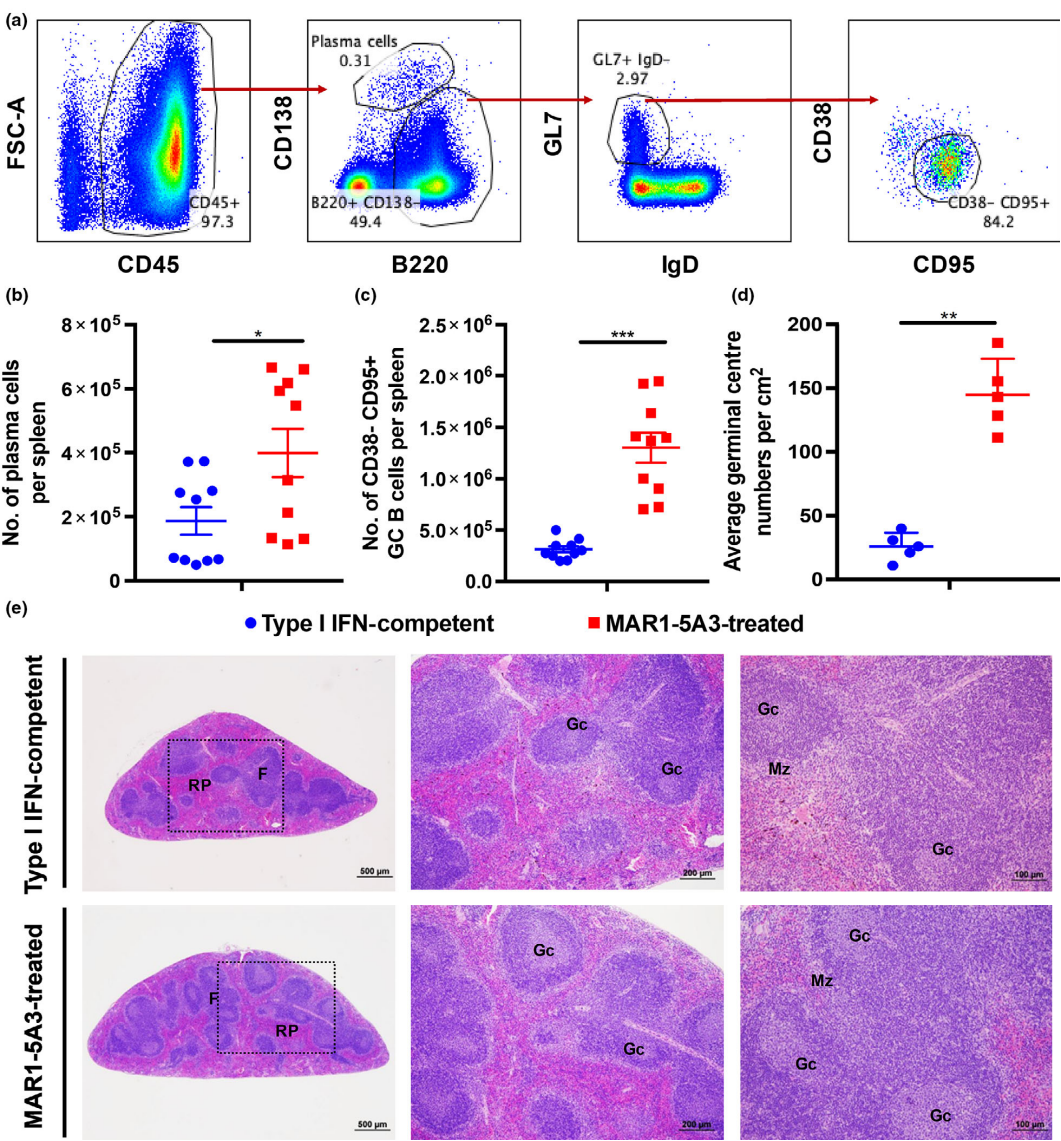
Formation of germinal centres in the mouse spleen is independent of type I IFN response during ZIKV infection at 12 dpi. ZIKV‐infected type I IFN‐competent and MAR1‐5A3‐treated WT mice were inoculated with 1 × 10^6^ PFU ZIKV i.v. by the retro‐orbital route. Two mg of MAR1‐5A3 was administered i.p. on the same day as infection. **(a)** Representative fluorescence‐activated cell sorting gating strategy to determine specific B‐cell subsets. Absolute numbers of **(b)** CD138^+^ plasma cells and **(c)** CD38^‐^CD95^+^ germinal centre B cells in spleen from respective groups at 12 dpi. Data are represented as mean ± SEM of two independent experiments with five animals per group per experiment. Statistical significance was measured using the Mann–Whitney *U*‐test (***P < 0.001). **(d)** Average germinal centre numbers were calculated from four different pieces from each spleen tissues (*n* = 5 animals/group). Data are represented as mean ± SD. Statistical significance was measured using the Mann–Whitney *U*‐test (**P < 0.01). **(e)** Representative photomicrograph of H&E‐stained section of germinal centres in spleen showed an increase in absolute number of germinal centre in MAR1‐5A3‐treated mice (bottom) than type I IFN‐competent mice (top). Dotted box indicates blown up regions. RP, red pulp; F, lymphoid follicles; GC, germinal centre; MZ, marginal zone on histological images of spleen sections (*n* = 5 animals/group).

Next, we assessed whether the differences in IgG levels was reflected in the germinal centres.^24^ Flow cytometry was performed to investigate B‐cell subsets in the spleen (Figure 2a). As expected, an increase in the presence of CD138^+^ plasma cells (Figure 2b) and CD38^‐^CD95^+^ germinal centre B cells (Figure 2c) was observed in MAR1‐5A3‐treated animals compared to controls. These observations were further validated histologically, with a visible increase in germinal centre in the white pulp of mouse spleen in MAR1‐5A3‐treated animals (Figure 2d and e). Together, these results show that type I IFN response suppression increases the amount of antibody response during ZIKV infection through an increase in germinal centres and plasma cells without affecting antibody class switching.

### Type I IFN suppression does not alter serum neutralising capacity

**Figure 3:**
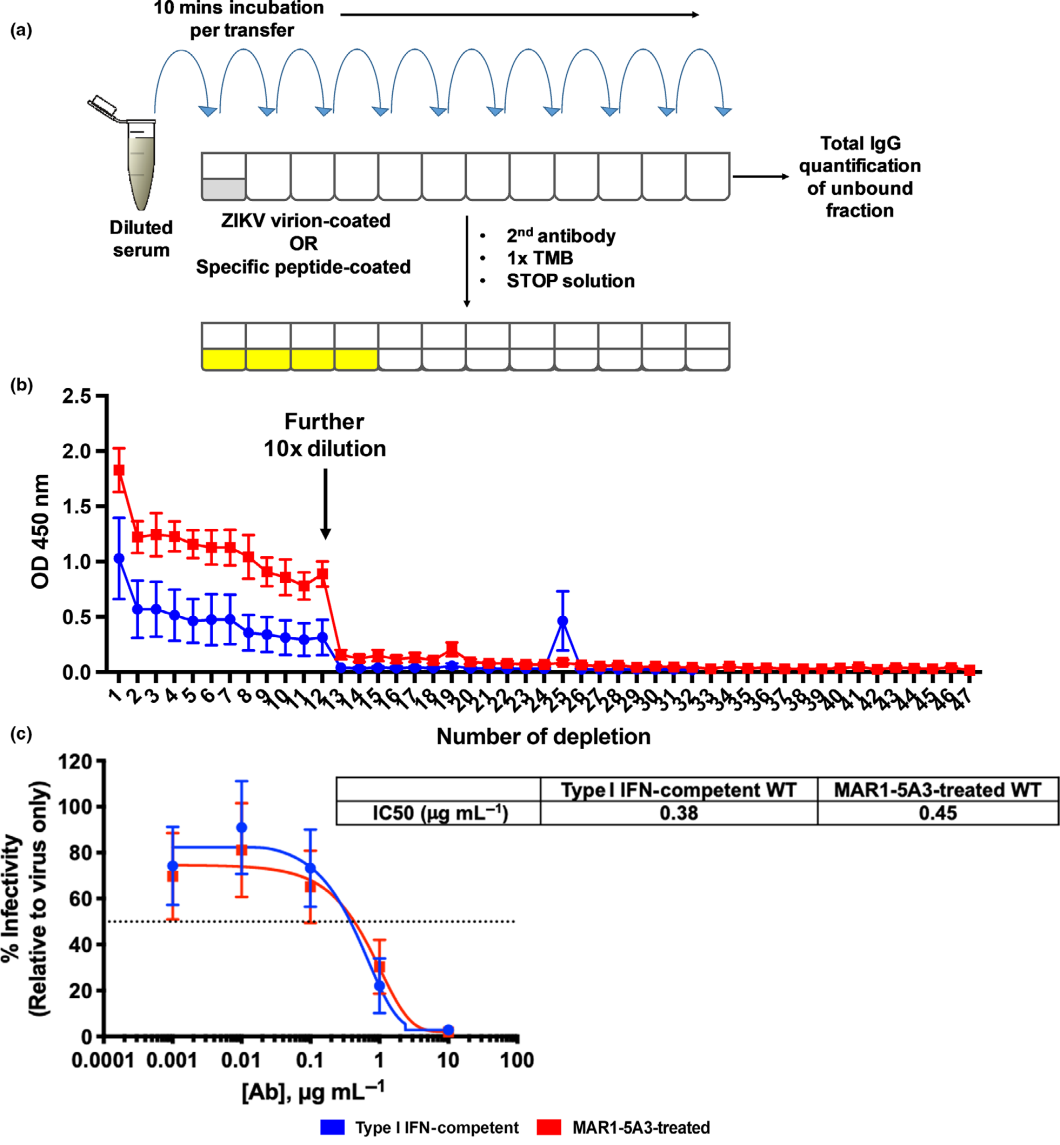
Type I IFN suppression does not alter the neutralising capacity of ZIKV‐specific antibodies. **(a)** Schematic diagram on ZIKV‐specific antibody depletion assay. **(b)** Verification of complete ZIKV‐specific IgG depletion. Pooled mouse sera samples (*n* = 5 animals per group) at 1:500 were added onto ZIKV virion‐coated 96‐well plates and incubated for 10 min per transfer at room temperature for adsorption. A further 10× dilution to 1:5000 was done at the 12th well to shorten the depletion process. The unbound portion was collected after 47 rounds of adsorption for total IgG quantification. ZIKV‐specific IgG concentration was obtained by subtracting the total IgG concentration in ZIKV‐specific IgG‐depleted serum from the total IgG concentration in non‐depleted serum. **(c)** Neutralising capabilities of type I IFN‐competent and MAR1‐5A3‐treated WT mice serum *in vitro*. ZIKV was pre‐incubated with 10‐fold serial dilutions of 45 dpi ZIKV‐specific IgG prior to infecting HEK 293T cells at MOI 10. Mock‐infected and ZIKV only conditions were used as controls. Infectivity was quantified 48 h post‐infection by immunofluorescence. Data are normalised to ZIKV only control and represent an average of two independent experiments. Nonlinear regression fitting was used to determine the IC50 values.

We next investigated whether antibody quantities would impact the neutralisation capacity against ZIKV infection. In order to mitigate any biasness, anti‐ZIKV IgG were normalised to the same concentrations prior to performing downstream comparative assays. As there are no commercially available assays to assess the absolute amount of ZIKV‐specific antibodies, quantification was performed by calculating the difference in antibody levels between neat sera and ZIKV‐depleted sera, similar to an earlier study^25^ (Figure 3a and b and Supplementary table 1). This approach was used as it does not encounter the issues of slope differences, which is inevitable during single‐point interpolation of commercial capture ELISA kits.^26^ Prior to performing neutralisation assays, we validated that our HEK293T cell line was susceptible to ZIKV infection (Supplementary figure 2). In agreement with other studies, ZIKV is able to infect and replicate in HEK293T cell line, with an observable infection and replication (Supplementary figure 2).^27^, ^28^ Interestingly, sera normalised to the same concentrations of ZIKV‐specific IgG showed similar neutralising capacity with EC50 of 0.38 and 0.45 μg mL^−1^, respectively, and a complete neutralisation of ZIKV infection at 10 μg mL^−1^ (Figure 3c). To validate this result using more standard techniques, a plaque reduction neutralisation test (PRNT) using VeroE6 cells was performed, which showed a similar profile (Supplementary figure 3a). Together, these data revealed that higher IgG production in mice with suppression of type I IFN did not result in a higher neutralising capability, suggesting that a sustained infection did not lead to an increased affinity maturation.

### Dominant epitopes are different on ZIKV E and NS1

Although there was no difference on virus neutralisation in MAR1‐5A3‐treated group at similar anti‐ZIKV antibody concentration, we investigated whether type I IFN inhibition altered the antigenic/epitope repertoire. Given that pre‐membrane (prM) protein, envelope (E) glycoprotein and non‐structural protein 1 (NS1) are the major antigenic targets during *flavivirus* infections,^29^, ^30^, ^31^ a panel of K562 cell lines transduced to express the ectodomain of ZIKV E, full‐length ZIKV prM and ZIKV NS1 on their cell surface generated previously^18^ was used to determine the antigenic targets. Each transduced cell line and control untransduced K562 cells were incubated with either 0.5 or 1 μg mL^−1^ IgG of pooled sera from type I IFN‐competent and MAR1‐5A3‐treated animals, followed by detection with a fluorescent secondary antibody. K562 cell surface displaying ZIKV E ectodomain and NS1 (but not prM) was clearly bound by both groups of sera (Supplementary figures 4 and 5), showing that they have the same antigenic targets.

**Figure 4:**
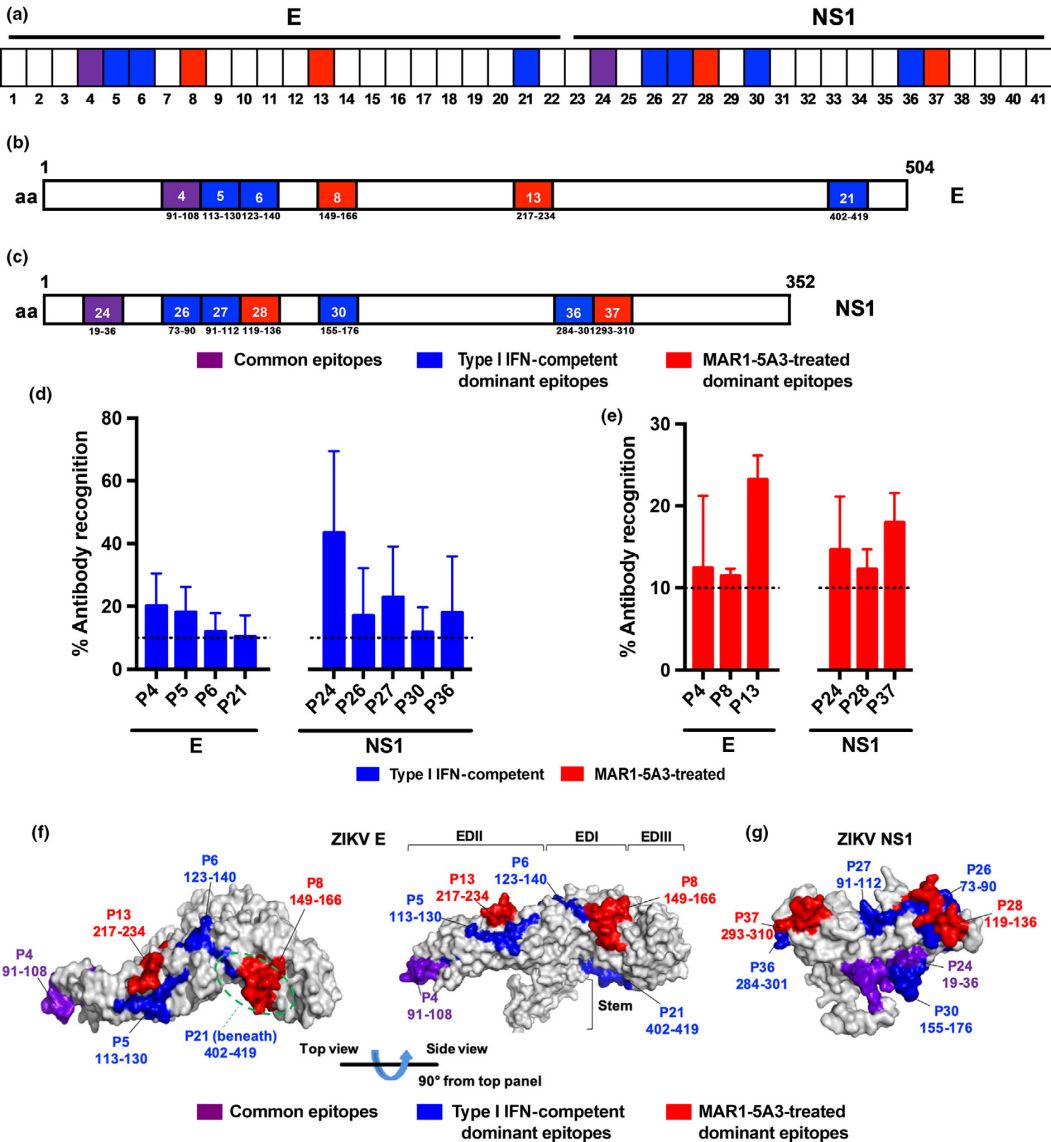
Mapping of ZIKV B‐cell linear epitopes within the ZIKV proteome. **(a–e)** Pooled sera at 45 dpi from type I IFN‐competent (*n* = 5) and MAR1‐5A3‐treated (*n* = 5) WT mice were tested at 1:250 by peptide‐based ELISA, using peptides that cover the E (peptides 1–22) and NS1 (peptides 23–41) of the ZIKV proteome. **(a)** A schematic representation to denote type I IFN‐competent dominant (in blue), MAR1‐5A3‐treated dominant (in red) and common (in purple) peptides across E and NS1. Regions of amino acids corresponding to the identified B‐cell linear epitopes in **(b)** ZIKV E and **(c)** NS1 are shown. Numbers in boxes denote the peptide number, and the amino acid position in the respective proteome. **(d)** Peptides plotted are those that are highly recognised (> 10%) by ZIKV‐specific antibodies from type I IFN‐competent or **(e)** MAR1‐5A3‐treated groups. Data are presented as mean ± SEM of three independent experiments with five animals per group per experiment. Percentage of antibody recognition was calculated according to this equation: % antibody recognition = 100 × (OD values from individual peptide group/sum of OD values from all peptide groups within the same antigen). Localisation of highly recognised ZIKV B‐cell linear epitopes on **(f)** ZIKV E monomer and **(g)** NS1 monomer based on the structural data retrieved from PDB records: 5IZ7 and 5K6K, respectively. Peptide regions recognised by type I IFN‐competent WT mice sera are in blue, while those of MAR1‐5A3‐treated WT mice sera are in red. Commonly recognised peptides are denoted in purple.

Next, specific peptide regions recognised by anti‐ZIKV antibodies were assessed using a peptide‐based ELISA of individual linear peptides of ZIKV E and NS1 regions (Supplementary table 2). Recognition of specific peptide regions was expressed as percentage of antibody recognition within each individual antigen and was further categorised as: highly recognised (> 10%), moderate (> 5%), low (> 1%) and no binding (< 1%). Interestingly, the two groups of sera bound predominantly to different peptide regions of both antigens (Table 1 and Supplementary table 3). For highly recognised (immuno‐dominant) epitopes (Figure 4a), type I IFN‐competent sera preferentially bound to peptides P5, P6 and P21 of the ZIKV E protein, and peptides P26, P27, P30 and P36 of the ZIKV NS1 protein (Figure 4b and c). Sera from the MAR1‐5A3‐treated group preferentially recognised peptides P8 and P13 of the ZIKV E protein, and peptides P28 and P37 of the ZIKV NS1 protein (Figure 4b and c). In addition, peptides P4 and P24 of the ZIKV E and NS1 proteins, respectively, were highly recognised by both groups (Figure 4b and c). All highly recognised epitopes each has a differential percentage of antibody recognition (Figure 4d and e). Taken together, these data show that suppression of type I IFN signalling can impact the B‐cell repertoire of dominant epitopes during ZIKV infection. The localisation of these dominant epitopes within the viral proteins is shown in Figure 4f and g. Interestingly, majority of the ZIKV E dominant epitopes are located on the exterior of the glycoprotein, with the exception of peptide P21, which is located at the bottom of the protein (Figure 4f), suggesting that these regions could be recognised *in vivo* and antibodies targeting these epitopes may be potentially neutralising.

**Table 1:** Differential recognition levels against B‐cell linear epitopes on the ZIKV E protein between type I IFN‐competent and MAR1‐5A3‐treated WT mice sera

Type I IFN‐competent		MAR1‐5A3‐treated		Recognition level
Recognition (%)	Peptide	Recognition (%)	Peptide	
20.10431766	P4	23.24116282	P13	High (> 10%)
18.09792046	P5	12.45284851	P4	
11.93779460	P6	11.45995791	P8	
10.40083997	P21			
8.323439586	P22	7.394968251	P9	Moderate (> 5%)
7.680286762	P8	7.189214007	P5	
6.108796096	P12	6.539186394	P22	
5.419987628	P13	5.538718881	P10	
4.285099050	P14	3.667888723	P14	Low (> 1%)
1.710600854	P9	3.059489052	P21	
1.657025721	P7	3.049874764	P3	
		2.463014208	P6	
		2.187464166	P7	
		1.709177371	P12	
		1.486916947	P2	
		1.393063199	P15	
		1.278410580	P17	
		1.186054052	P20	
		1.157335812	P18	
		1.132520757	P16	
0.980205068	P3	0.786510690	P1	No binding (< 1%)
0.856522941	P15	0.714034207	P19	
0.825107816	P16	0.627313444	P11	
0.788987195	P18			
0.747653521	P20			
0.735128394	P1			
0.580044173	P2			
0.555611431	P10			
0.442519394	P19			
0.290453251	P11			
0	P17			

### Dominant epitopes may not necessarily be important for virus neutralisation

**Figure 5:**
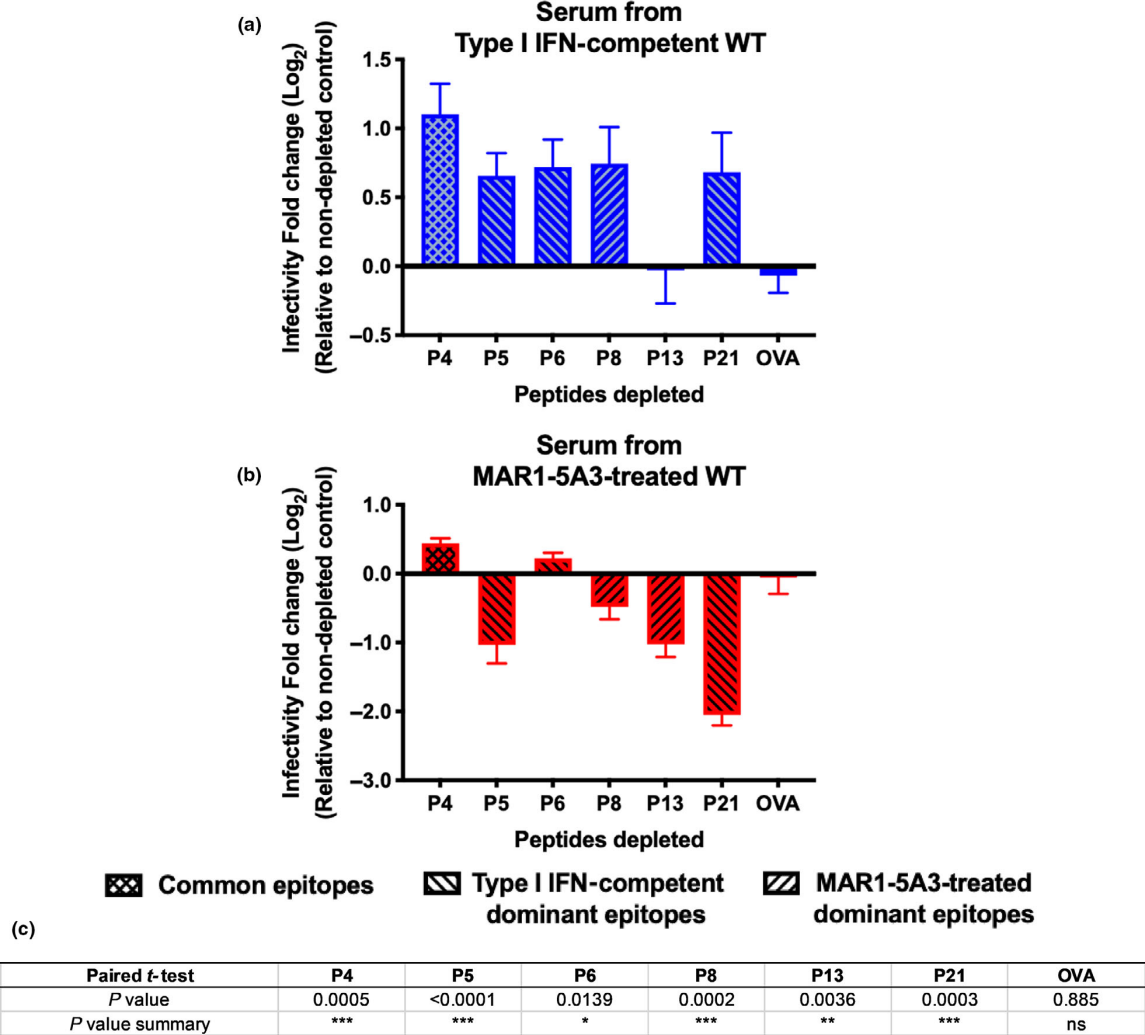
Antibodies highly recognising ZIKV E epitopes have a non‐specific role during ZIKV infection. Neutralising capability of pooled mouse sera from **(a)** type I IFN‐competent or **(b)** MAR1‐5A3‐treated WT animals upon depletion of ZIKV‐specific antibodies targeting peptides that are either common epitope: P4, type I IFN‐competent dominant epitopes: P5, P6 or P21, or MAR1‐5A3‐treated dominant epitopes: P8 or P13. A non‐specific peptide control, OVA, was also included. Neutralisation assays were performed at 0.5 µg mL^−1^ of ZIKV‐specific IgG. Data are represented as mean ± SEM of two independent experiments with five animals per group per experiment. Results are expressed as log_2_ fold change relative to the respective non‐depleted controls. **(c)** A two‐tailed paired *t*‐test analysis on the neutralising capability of pooled mouse sera from either type I IFN‐competent or MAR1‐5A3‐treated animals after depletion of ZIKV‐specific antibodies targeting peptides P4, P5, P6, P8, P13, P21 and OVA.

We next investigated whether antibodies targeting these dominant epitopes were neutralising and could therefore be potential therapeutic candidate targets for vaccine or monoclonal antibody design. Since NS1 is not part of the viral particle, only dominant epitopes of the ZIKV E protein were tested. Antibodies targeting dominant B‐cell linear epitopes were depleted individually, and depletion was validated by ELISA (Supplementary figure 6a–d). The peptide‐depleted sera were then tested at a fixed concentration of 0.5 μg mL^−1^ of ZIKV‐specific IgG for their *in vitro* neutralisation capacity against ZIKV. Depletion of antibodies targeting dominant epitopes for the type I IFN‐competent group resulted in higher infectivity level relative to their respective non‐depleted control (Figure 5a), indicating the importance of antibodies against these regions in neutralising ZIKV infection. For sera collected from MAR1‐5A3‐treated animals, only the common epitope (P4) and a dominant epitope from the type I IFN‐competent group (P6) showed neutralisation capacity albeit lower than the type I IFN‐competent sera (Figure 5b and c). Surprisingly, sera from MAR1‐5A3‐treated group depleted of the other peptides (P5, P8, P13 or P21) showed a significant decrease in ZIKV infection, suggesting a detrimental effect of the antibodies targeting these epitopes (Figure 5b and c). These data were confirmed by PRNT assay using VeroE6 cells (Supplementary figure 3b and c). In addition, depletion of peptide‐specific antibodies did not result in a significant change towards binding to ZIKV virions, indicating that the antibodies targeting ZIKV B‐cell linear epitopes are present at low levels (Supplementary figure 6e and f).

To determine whether these detrimental effects could be due to peptide‐specific IgG subtypes, the proportion of various IgG subtypes was assessed by ELISA (Supplementary figure 6g). Interestingly, while the type I IFN‐competent group showed a high diversity in IgG subtype proportions with more IgG1 fraction for type I IFN‐competent dominant peptides, the MAR1‐5A3‐treated group showed a majority of IgG2c isotype for all the peptides tested (Supplementary figure 6g). These results suggest that type I IFN suppression may trigger a detrimental anti‐ZIKV IgG response, which is compensated by higher levels of anti‐ZIKV total IgG levels (Figure 1c).

## Discussion

Type I IFN signalling has been closely associated with direct antiviral responses, and recent findings have also highlighted type I IFNs as a potential regulator for adaptive immunity, including humoral responses.^15^, ^32^ In this study, 2 mg of MAR1‐5A3 anti‐IFNAR1 antibody was administered into immunocompetent WT animals on the same day as infection. Given that the half‐life of 2 mg MAR1‐5A3 is approximately 5.2 days,^11^ we surmise that this condition mimics the transient suppression of type I IFN responses by ZIKV reported in patients.^12^, ^13^ In addition, a single large bolus of MAR1‐5A3 is usually administered in order to sufficiently saturate the IFNAR‐1 receptor pool.^11^, ^33^, ^34^, ^35^, ^36^, ^37^, ^38^ MAR1‐5A3 transiently blocks IFNAR‐1 receptors and does not deplete any IFNAR‐1 receptors or circulating IFNα/β. More importantly, administration of MAR1‐5A3 does not induce any antibody response, suggesting that there are no confounding factors contributed by MAR1‐5A3 towards the anti‐ZIKV humoral response.^11^

Type I IFN‐competent animals displayed persistent anti‐ZIKV IgM levels while these levels declined in MAR1‐5A3‐treated mice. This prolonged IgM response in WT animals is consistent with clinical studies where patient IgM levels remained detectable up to a year following flaviviral infections.^23^, ^39^, ^40^ Several studies have associated type I IFN signalling in dendritic cells in promoting virus‐specific IgM,^14^, ^41^ which could explain our observations.

In both groups, IgG response increases through the course of infection, with a higher ZIKV‐specific IgG level when type I IFN was suppressed. This observation is consistent with the increased number of CD138^+^ plasma cells and CD38^‐^CD95^+^ germinal centre B cells present in the spleen of MAR1‐5A3‐treated animals, which is further supported by an increase in germinal centres. It is plausible that the increased B‐cell response could be due to an increased abundance of ZIKV antigens following an active viral replication in MAR1‐5A3‐treated animals, which would be similar to effects observed during chronic infections where viral persistence led to virus‐specific antibody inflation.^42^ In addition, it could also be due to a compensatory effect when the type I IFN is inactivated, resulting in other pathways, in this case, the humoral response, to be more activated.^43^ Levels of murine IgG3 directed against ZIKV were undetectable, consistent with IgG3 being mostly induced by carbohydrate‐ and T‐independent antigens.^44^, ^45^, ^46^ During acute ZIKV infection, IgG1 was the dominant subclass while IgG2c was the dominant IgG subtype at later time points in both groups. This suggests that antibody class switching is not altered upon type I IFN suppression and that IgG2c is the dominant subclass produced during anti‐ZIKV humoral response, consistent with studies on other viral infections in mice.^44^, ^46^, ^47^

Most protective antibodies are often directed against flaviviral prM, E and NS1 proteins^31^, ^47^; however, surprisingly, prM was not one of the major targets in this study. Importantly, mechanisms underlying *flavivirus* immunity and immunopathology may be attributed towards differences in determinant recognition, thereby leading to differential protection or production of neutralising antibodies.^31^ Notably, type I IFN suppression may have influenced the determinant recognition of the antibodies as there is a vast difference in dominant epitope numbers and regions on both the ZIKV E and NS1 proteins between both groups. Although the involvement of type I IFN signalling towards B‐cell repertoire diversity remains largely undefined, this phenomenon could be a resultant of enhanced follicular B‐cell activities promoted by type I IFN signalling, where more diverse repertoires are expressed.^48^, ^49^ In both groups of sera, the loss of antibodies targeting common peptide P4 (residues 91–108) of the ZIKV E protein resulted in an enhanced ZIKV infection. This is expected as P4 is in close proximity to the fusion loop within EDII, which has been reported as a region targeted by potent flaviviral neutralising antibodies.^50^, ^51^

All the ZIKV E dominant epitopes (with the exception of peptide P13) have an antiviral role in the immunocompetent animals. Intriguingly, an opposite effect was observed in the MAR1‐5A3‐treated group, where all the dominant epitopes (except peptide P6) are non‐neutralising against ZIKV infection. This observation is accompanied by a lack of differences towards the binding of ZIKV virions after peptide‐specific antibody depletion, suggesting that antibodies targeting linear epitopes are present in minority, albeit having an impact towards ZIKV neutralisation. It is of immunological interest that B‐cell linear epitope specificity between both groups gave rise to opposing immune responses (Figure 5); thus, studies to assess conformational epitopes remain imperative. While it remains elusive, the presence of antibodies targeting B‐cell linear epitopes in the MAR1‐5A3‐treated animals may be hindering other potentially neutralising antibodies, and specific mechanistic effects of these epitope‐specific antibodies would require the generation of monoclonal antibodies. However, a defect in the innate effector mechanism could result in an enhanced adaptive immune response, which may be detrimental to the host.^43^ In addition, one of the consequences of an increased amounts of antibodies induced during a viral infection is dysregulation of the feedback mechanism of the immune system.^52^ Nonetheless, looking into the peptide‐specific IgG subtypes further revealed a difference in IgG proportions between both groups, with IgG2c being the majority subclass when type I IFN is suppressed. However, it remains to be further explored whether such observations could be attributed by the differing proportions of IgG subtypes, in which the constant region of an antibody can greatly affect the variable region structure, which translates into differences in affinity and/or specificity.^53^ Interestingly, it has been previously reported that type I IFN induces IgG2c class switching,^47^, ^54^ which did not appear impaired in this study against ZIKV. This suggested that subtype switching could be type I IFN independent and driven by other factors, such as cognate interactions between T and B cells.^55^ Furthermore, it is known that humoral response including B‐cell proliferation, antibody production and immunoglobulin class switching is dependent on T cells help.^56^, ^57^ While it is not investigated in this work, it would be important to assess the potential effects of type I IFN suppression towards T‐cell response, in particular, the balance between Th1 and Th2 activities in modulating the host humoral response during ZIKV infection.^56^ In addition, it would also be critical to study the effects of type I IFN blockade on follicular dendritic cells and T follicular helper (Tfh) cells, especially since it has been reported in a recent study where Tfh cells regulate the magnitude and quality of the anti‐ZIKV antibody response.^58^, ^59^

Although antibodies targeting dominant epitopes of the ZIKV NS1 protein were not tested for their functional activity in this study as they do not directly neutralise virus infectivity, their protection by other effector mechanisms has been vastly reported.^31^, ^60^ As such, the dominant NS1 regions that were identified here could be further explored for their potential involvement towards controlling *flavivirus* infections.

In light of our data shown here, it could be aligned with other *flavivirus* pathogenesis, since suppression of the type I IFN response is an immune evasion mechanism employed by *flaviviruses* including ZIKV and dengue virus (DENV).^12^, ^13^, ^61^, ^62^, ^63^ It could be possible that generation of non‐protective antibodies is hindering the accessibility and binding of truly neutralising antibodies, thereby contributing to exacerbation of disease severity. Of clinical importance, ZIKV infection has been associated with Guillain–Barré syndrome, an autoimmune disease involving autoantibodies targeting the gangliosides.^64^ Thus, it would be crucial to elucidate any possible association, especially since type I IFN has a role in regulating autoantibodies' production.^65^

## Methods

### Virus

Zika virus Polynesian isolate ZIKV H/PF/2013 was obtained from the European Virus Archive (EVA, Marseille, France). Virus was propagated in VeroE6 cells (ATCC, Manassas, VA, USA) or in C6/36 mosquito cells (ATCC) for infection studies. Virus stocks were purified via ultracentrifugation and titred by standard plaque assays in VeroE6 cells as previously described.^18^

### Mice

Four‐week‐old C57BL/6 WT mice were bred and kept under specific pathogen‐free conditions in the Biological Resource Centre, Agency of Science, Technology and Research, Singapore. All mouse studies were approved by the Institutional Animal Care and Use Committee (IACUC: 181353) of the Agency for Science, Technology and Research, Singapore (A*STAR), performed according to the guidelines of the Agri‐Food and Veterinary Authority and the National Advisory Committee for Laboratory Animal Research of Singapore.

### Virus inoculation and antibody administration

Mice were inoculated intravenously (i.v.) through the retro‐orbital route with 1 × 10^6^ PFU ZIKV in 100 μL PBS. Blockade of type I interferon (IFN) was done by administration with two doses of 1 mg of mouse IgG1 anti‐IFN alpha/beta receptor 1, MAR1‐5A3 (Leinco Technologies, St. Louis, MO, USA)^11^ antibody intraperitoneally (i.p.) on the same day as infection.

### Viral RNA extraction and quantification

Viraemia was monitored on 1, 2, 3, 4, 7, 12 and 45 days post‐infection (dpi). Ten microlitres of blood collected from the tail vein was diluted in 120 μL of PBS and 10 μL citrate‐phosphate‐dextrose solution (Sigma‐Aldrich, St. Louis, MO, USA). Viral RNA was extracted with the QIAamp Viral RNA Kit (Qiagen, Hilden, Germany) following the manufacturer's protocol. NS5 RNA copies were quantified in 1 μL of viral RNA sample by quantitative reverse transcription PCR (qRT‐PCR) using QuantiTect Probe RT‐PCR Kit (Qiagen) as previously described.^66^

### Serum processing

Blood was collected from the animals through retro‐orbital bleeding using glass capillary tubes (Fisherbrand, Waltham, MA, USA). Collected blood was left to clot at RT for at least an hour prior to centrifuging at 9 400 *g* for 5 min. Centrifugation was done twice to obtain clot‐free serum. Samples were then heat‐inactivated at 56°C for 30 min and cooled on ice for at least 30 min before storing at −20°C or downstream serological assays. Serum samples were obtained from three different batches of animals per group (Supplementary table 1).

### Semi‐quantification of ZIKV‐specific antibody titre and IgG subtyping

Antibody titres were determined by virion‐based ELISA as previously described.^18^, ^19^ Pooled heat‐inactivated mouse sera of respective groups were tested at 1:100 dilution for IgM, and 1:500 dilution for IgG. For IgG subtyping studies, pooled sera were serially diluted from 1:250 to 1:32 000 for IgG3, 1:512 000 for IgG1 and IgG2b, and 1:8 192 000 for IgG2c. ZIKV‐specific IgG subtypes are expressed as antibody titre, defined as the greatest reacting dilution before the OD value reaches baseline control (pooled 0 dpi sera). HRP‐conjugated goat anti‐mouse IgM (Santa Cruz Biotechnology, Dallas, TX, USA), IgG (Merck, Kenilworth, NJ, USA), IgG1 (Santa Cruz Biotechnology), IgG2b (Santa Cruz Biotechnology), IgG2c (Southern Biotech, Birmingham, AL, USA) and IgG3 (Santa Cruz Biotechnology) antibodies were used. IgG2c was tested in lieu of IgG2a since only IgG2c gene is present in C57BL/6 mice.^47^, ^67^ ELISAs were developed using TMB substrate and terminated with stop reagents (Sigma‐Aldrich). The absorbance was measured at 450 nm. ELISA readings were conducted in duplicates.

### ZIKV‐specific IgG quantification

Quantification of antigen‐specific antibodies by depletion was adapted from Lemke *et al*., 2004. Briefly, depletion of anti‐ZIKV antibodies was done using ZIKV virion‐coated (1 × 10^6^ virions per well) 96‐well maxisorp microtitre plates (Nunc, Roskilde, Denmark). Pooled mouse sera samples from 45 dpi were added at 1:500 and incubated for 10 min at room temperature for adsorption. A further 10 × dilution to 1:5000 was done at the 12th well to shorten the depletion process. The unbound portion was collected after 47 rounds of adsorption. ELISA analysis was performed to verify complete depletion of ZIKV‐specific antibodies. IgG concentrations were then quantified using an IgG Mouse ELISA kit (Abcam, Cambridge, UK) according to the manufacturer's protocol where serum samples were recommended to dilute at 10 000×. ZIKV‐specific IgG concentration was obtained by subtracting the total IgG concentration in ZIKV‐specific IgG‐depleted serum from the total IgG concentration in non‐depleted serum (Supplementary table 1).

### Identification of ZIKV antigen target

Using a protocol as previously described,^18^ transduced K562 cell lines with over 90% surface expression of either ZIKV E ectodomain (with the transmembrane region deleted), prM or NS1 were incubated with 0.1, 0.5 or 1.0 μg mL^−1^ of respective pooled mouse serum for 30 min at room temperature prior to labelling with a fluorophore‐tagged secondary goat anti‐mouse IgG (H+L) antibody (Life Technologies, Carlsbad, CA, USA). Cell nuclei were labelled with DAPI for analysis by flow cytometry.

### Epitope determination and structural localisation

Peptide‐based ELISA was performed as previously described^18^, ^19^ to screen for viral epitopes using a library of high purity biotinylated peptides (≥ 90%, EMC microcollections GmbH, Tuebingen, Germany). Lengths consisting of 11 to 22‐mer peptides were generated from ZIKV Polynesian isolate (KJ776791). Peptides were dissolved in DMSO (Sigma‐Aldrich) to obtain a stock concentration of 3.75 μg μL^−1^. Diluted biotinylated peptides were added to pre‐blocked streptavidin plates (Thermo Fisher Scientific, Waltham, MA, USA), and sera samples (at 1:250 dilution) and HRP‐conjugated anti‐mouse IgG (Merck) were used. All the peptide samples were screened in triplicates using normalised 45 dpi pooled sera from respective groups of mice, with 0 dpi sera as a baseline control. Percentage of antibody recognition on different ZIKV proteins was calculated according to this equation: % antibody recognition = 100 × (OD values from individual peptide group/sum of OD values from all peptide groups within the same antigen). This approach would enable the identification of similar and/or different target epitopes elicited upon type I IFN blockade based on the percentage range, which will be further categorised into different recognition levels (high, moderate, low or no binding). Structural data of ZIKV E glycoprotein and NS1 were retrieved from Protein Databank (PDB IDs 5IZ7 and 5K6K, respectively), and visualised using PyMOL (Schrodinger, version 2.2.0, Cambridge, MA).

### ZIKV infection kinetic on HEK293T cells

Zika virus infection on HEK293T cell lines was performed at MOI 1 and 10. Cells were seeded at 8 × 10^5^ cells per 60 mm^2^ dishes in complete Dulbecco's modified Eagle's medium (DMEM; GE Healthcare Life Sciences, Pittsburgh, PA, USA) supplemented with 10% foetal bovine serum (FBS; GE Healthcare Life Sciences) 1 day prior to infection. Virus infection mix is composed of virus suspension prepared in serum‐free DMEM. Virus overlay was incubated with seeded cells at 37°C for 2 h with intermittent rocking. After adsorption, virus overlay was aspirated and replenished with complete media. Mock infections (without virus) were also performed in parallel as controls. Cells were then incubated at 37°C until time of harvest at different time points. During harvesting, 140 μL of infected cell suspension was collected for viral RNA extraction. Remaining cells were collected by centrifugation and stained with live/dead dye (Life Technologies) before fixing with 1× FACS lysing buffer (BD Biosciences, Franklin Lakes, NJ, USA), and kept at 4°C before assessing for infection by flow cytometry.

### Flow cytometry

Fixed cells were permeabilised with 1× FACS permeabilising solution 2 (BD Biosciences). Quantification of ZIKV infectivity was done through the detection of ZIKV antigens by performing a 2‐step staining procedure using rabbit anti‐ZIKV NS3^68^ as the primary antibody, followed by secondary staining with Alexa Fluor 647‐conjugated goat anti‐rabbit IgG (Thermo Fisher Scientific). Samples were acquired using LSRFortessa (BD Biosciences) with BD FACSDIVA™ software.

### Affinity depletion of selected anti‐ZIKV antibodies

Selected synthetic biotinylated peptides (EMC microcollections GmbH) were added at 375 ng/well to streptavidin‐coated plates (Thermo Fisher Scientific) and incubated at room temperature for 1 h in PBS containing 0.1% Tween‐20 (0.1% PBST). Pooled mouse sera samples were prepared to a concentration of 20 μg mL^−1^ of ZIKV‐specific IgG, and 50 μL per well of samples was added and incubated for 10 min at room temperature for adsorption. The unbound portion was collected after 16 rounds of adsorption. ELISA analysis was performed to verify the levels of selected antibodies after affinity depletion. Depleted samples were then mixed with ZIKV at a multiplicity of infection (MOI) of 10, and incubated for 2 h at 37°C with gentle agitation at 350 rpm. Sero‐neutralisation assay was performed to verify the neutralising activity.

### Sero‐neutralisation assay

Neutralising activity of respective groups of pooled mouse sera was tested in triplicates and analysed by immunofluorescence‐based cell infection assay in HEK 293T cells. ZIKV was mixed with diluted serum to obtain an MOI of 10. This virus‐serum mix was incubated for 2 h at 37°C with gentle agitation at 350 rpm. Virus‐serum mixtures were then added to HEK 293T cells seeded in 96‐well plates and incubated for 2 h at 37°C. The mixture overlay was removed, and cells were replenished with complete DMEM (GE Healthcare Life Sciences), and incubated for 48 h at 37°C before staining with live/dead dye (Life Technologies). Cells were then fixed with 4% paraformaldehyde (Electron Microscopy Sciences, Hatfield, PA, USA) followed by permeabilisation with staining buffer (3% BSA, 5% FBS, 0.1% PBST, 0.1% Triton X 100). Cells were stained with ZIKV NS3 protein‐specific rabbit polyclonal antibody^68^ for 1 h, followed by staining with a fluorophore‐tagged secondary goat anti‐rabbit IgG (H+L) antibody (Life Technologies) for 1 h before acquisition with MACSquant Analyser 10 (Miltenyi Biotec, Bergisch Gladbach, Germany) with MACSQuantify™ software.

### Plaque reduction neutralisation test (PRNT)

VeroE6 cells were seeded at 1 × 10^4^ cells/well in 24‐well tissue culture plates (Corning, New York, NY) 1 day prior to infection. ZIKV was diluted in cell culture media to yield 60–120 plaques/well in the virus control wells. Pooled serum samples were 5‐fold serially diluted. An equal volume of ZIKV was added to each diluted serum sample, and the virus‐serum mixture was incubated for 2 h at 37°C with gentle agitation at 350 rpm. Virus‐serum mixtures were then added to VeroE6 cells and incubated for 1 h at 37°C. After adsorption, 1 mL of 1% low melting point agarose (Promega, Madison, WI, USA) and 1× MEM mixture (Sigma‐Aldrich) was added to each well. Plates were left at room temperature to allow agarose overlay to set before incubating at 37°C for 4 days. A solution of neutral red was prepared on the day of use by adding 4 mL of 0.33% sterile neutral red (Sigma‐Aldrich) per 36 mL of PBS. On the day before completion of assay (day 3), 1 mL of neutral red solution was added to each well. The next day, the agarose‐neutral red overlay was aspirated from each well, and plates were blotted dry before counting the plaques.

### B cells profiling in the spleen

Spleens of mice were surgically extracted and dissociated in RPMI medium containing 10% FBS (complete RPMI) and passed through a 40 μm cell strainer (Fisherbrand), followed by RBC lysis with RBC lysis buffer (R&D system, Minneapolis, MN, USA). Isolated cells were stained with LIVE/DEAD Aqua (Life Technologies) and then incubated in 100 μL blocking buffer consisting of a mix of 1% rat and mouse serum (Sigma‐Aldrich) in FACS buffer (1% BSA, 2 μm EDTA in PBS). Next, cells were stained with conjugated antibodies for 20 min and fixed in IC fixation buffer (eBioscience, San Diego, CA, USA) for 5 min before acquisition using a LSR II flow cytometer (BD Biosciences, Franklin Lakes, NJ, USA). Conjugated antibodies used were CD45 (BD Bioscience), B220 (eBioscience), GL‐7 (BD Biosciences), CD95 (BD Biosciences), CD38 (Biolegend, San Diego, CA, USA), CD73 (eBioscience), CD138 (BD Bioscience) and IgD (BD Bioscience).

### Morphometric analysis of germinal centres

Mice were euthanised at 12 dpi, and spleens were harvested and fixed in 10% neutral buffered formalin. Fixed tissues were cut into four different pieces and were processed in paraffin. The tissues were then sectioned at 5 μm thickness and stained with H&E staining. Briefly, for morphometric analysis, the spleen was captured at 4× magnification using Nikon DS Fi3 camera fitted with Nikon 80i microscope. The total area of spleen was then measured using Nikon NIS element software. The total number of germinal centres in each spleen section was counted manually. The result was expressed as average number of GCs/cm^2^.

### Data and statistical analysis

Statistical analyses were performed using Prism 7 (GraphPad Software, San Diego, CA), using an unpaired nonparametric Mann–Whitney *U*‐test or two‐tailed paired *t*‐test. *P*‐values considered statistically significant are represented with * for *P* < 0.05, ** for *P* < 0.01, and *** for *P* < 0.001. Flow cytometry results were analysed with FlowJo (version 10.4.1, Tree Star Inc. Becton Dickinson, Ashland, OR).

## Conflict of interest

The authors declare no conflict of interest.

## Author contributions

CYPL and LFPN conceptualised the study. CYPL, ZYC, RR, THT and ATR performed the experiments. CYPL, GC, FML, FAB, LR and LFPN analysed the data. CYPL, GC, FML, RR, LR and LFPN wrote the manuscript. All other authors were involved in processing and analysis, and/or logistical support, and approved the final version of the manuscript.

## Supporting information

Fig S1

Fig S2

Fig S3

Fig S4

Fig S5

Fig S6

Table S1

Table S2

Table S3

## References

[1] Faye O , Faye O , Dupressoir A *et al* One‐step RT‐PCR for detection of Zika virus. J Clin Virol 2008; 43: 96–101. 18674965 10.1016/j.jcv.2008.05.005

[2] Musso D , Roche C , Robin E *et al* Potential sexual transmission of Zika virus. Emerg Infect Dis 2015; 21: 359–361. 25625872 10.3201/eid2102.141363 PMC4313657

[3] Besnard M , Lastere S , Teissier A , Cao‐Lormeau V , Musso D . Evidence of perinatal transmission of Zika virus, French Polynesia, December 2013 and February 2014. Euro Surveill 2014; 19: 20751. 24721538

[4] Miner JJ , Sene A , Richner JM *et al* Zika virus infection in mice causes panuveitis with shedding of virus in tears. Cell Rep 2016; 16: 3208–3218. 27612415 10.1016/j.celrep.2016.08.079 PMC5040391

[5] Mlakar J , Korva M , Tul N *et al* Zika virus associated with microcephaly. New Engl J Med 2016; 374: 951–958. 26862926 10.1056/NEJMoa1600651

[6] Lee CY , Ng LFP . Zika virus: from an obscurity to a priority. Microbes Infect 2018; 20: 635–645. 29530643 10.1016/j.micinf.2018.02.009

[7] Lazear HM , Govero J , Smith AM *et al* A mouse model of Zika virus pathogenesis. Cell Host Microbe 2016; 19: 720–730. 27066744 10.1016/j.chom.2016.03.010 PMC4866885

[8] Anderson JF , Rahal JJ . Efficacy of interferon α‐2b and ribavirin against West Nile virus *in vitro* . Emerg Infect Dis 2002; 8: 107–108. 11749765 10.3201/eid0801.010252 PMC2730275

[9] Best SM , Mitzel DN , Bloom ME . Action and reaction: the arthropod‐borne flaviviruses and host interferon responses. Future Virol 2006; 1: 447–459.

[10] Rossi SL , Tesh RB , Azar SR *et al* Characterization of a novel murine model to study Zika virus. Am J Trop Med Hyg 2016; 94: 1362–1369. 27022155 10.4269/ajtmh.16-0111 PMC4889758

[11] Sheehan KC , Lai KS , Dunn GP *et al* Blocking monoclonal antibodies specific for mouse IFN‐α/β receptor subunit 1 (IFNAR‐1) from mice immunized by *in vivo* hydrodynamic transfection. J Interferon Cytokine Res 2006; 26: 804–819. 17115899 10.1089/jir.2006.26.804

[12] Grant A , Ponia SS , Tripathi S *et al* Zika virus targets human STAT2 to inhibit type I interferon signaling. Cell Host Microbe 2016; 19: 882–890. 27212660 10.1016/j.chom.2016.05.009 PMC4900918

[13] Kumar A , Hou S , Airo AM *et al* Zika virus inhibits type‐I interferon production and downstream signaling. EMBO Rep 2016; 17: 1766–1775. 27797853 10.15252/embr.201642627 PMC5283583

[14] Zhu J , Huang X , Yang Y . Type I IFN signaling on both B and CD4 T cells is required for protective antibody response to adenovirus. J Immunol 2007; 178: 3505–3510. 17339445 10.4049/jimmunol.178.6.3505

[15] Le Bon A , Schiavoni G , D'Agostino G *et al* Type I interferons potently enhance humoral immunity and can promote isotype switching by stimulating dendritic cells *in vivo* . Immunity 2001; 14: 461–470. 11336691 10.1016/s1074-7613(01)00126-1

[16] Fallet B , Narr K , Ertuna YI *et al* Interferon‐driven deletion of antiviral B cells at the onset of chronic infection. Sci Immunol 2016; 1: eaah6817. 27872905 10.1126/sciimmunol.aah6817 PMC5115616

[17] Moseman EA , Wu T , de la Torre JC , Schwartzberg PL , McGavern DB . Type I interferon suppresses virus‐specific B cell responses by modulating CD8^+^ T cell differentiation. Sci Immunol 2016; 1: eaah3565. 27812556 10.1126/sciimmunol.aah3565 PMC5089817

[18] Kam Y‐W , Lee CY‐P , Teo T‐H *et al* Cross‐reactive dengue human monoclonal antibody prevents severe pathologies and death from Zika virus infections. JCI Insight 2017; 2: 92428. 28422757 10.1172/jci.insight.92428 PMC5396524

[19] Kam Y‐W , Lum F‐M , Teo T‐H *et al* Early neutralizing IgG response to Chikungunya virus in infected patients targets a dominant linear epitope on the E2 glycoprotein. EMBO Mol Med 2012; 4: 330–343. 22389221 10.1002/emmm.201200213 PMC3376860

[20] Frumence E , Viranaicken W , Bos S *et al* A chimeric Zika virus between viral strains MR766 and BeH819015 highlights a role for E‐glycan loop in antibody‐mediated virus neutralization. Vaccines 2019; 7: 55. 10.3390/vaccines7020055 PMC6630725 31238493

[21] Rapti K , Louis‐Jeune V , Kohlbrenner E *et al* Neutralizing antibodies against AAV serotypes 1, 2, 6, and 9 in sera of commonly used animal models. Mol Ther 2012; 20: 73–83. 21915102 10.1038/mt.2011.177 PMC3255603

[22] Dong M , Zhang PF , Grieder F *et al* Induction of primary virus‐cross‐reactive human immunodeficiency virus type 1‐neutralizing antibodies in small animals by using an alphavirus‐derived *in vivo* expression system. J Virol 2003; 77: 3119–3130. 12584337 10.1128/JVI.77.5.3119-3130.2003 PMC149731

[23] Paz‐Bailey G , Rosenberg ES , Doyle K *et al* Persistence of Zika virus in body fluids ‐ final report. N Engl J Med 2018; 379: 1234–1243. 28195756 10.1056/NEJMoa1613108 PMC5831142

[24] Tam HH , Melo MB , Kang M *et al* Sustained antigen availability during germinal center initiation enhances antibody responses to vaccination. Proc Natl Acad Sci USA 2016; 113: e6639–e6648. 27702895 10.1073/pnas.1606050113 PMC5086995

[25] Lemke CD , Haynes JS , Spaete R *et al* Lymphoid hyperplasia resulting in immune dysregulation is caused by porcine reproductive and respiratory syndrome virus infection in neonatal pigs. J Immunol 2004; 172: 1916–1925. 14734777 10.4049/jimmunol.172.3.1916

[26] Barrette RW , Urbonas J , Silbart LK . Quantifying specific antibody concentrations by enzyme‐linked immunosorbent assay using slope correction. Clin Vaccine Immunol 2006; 13: 802–805. 16829619 10.1128/CVI.00422-05 PMC1489577

[27] Liu H , Liao HM , Li B *et al* Comparative genomics, infectivity and cytopathogenicity of American isolates of Zika virus that developed persistent infections in human embryonic kidney (HEK293) cells. Int J Mol Sci 2019; 20: 3035. 10.3390/ijms20123035 PMC6628096 31234341

[28] Ramos da Silva S , Cheng F , Huang IC , Jung JU , Gao SJ . Efficiencies and kinetics of infection in different cell types/lines by African and Asian strains of Zika virus. J Med Virol 2019; 91: 179–189. 30192399 10.1002/jmv.25306 PMC6294704

[29] Wahala WMPB , de Silva AM . The human antibody response to dengue virus infection. Viruses 2011; 3: 2374–2395. 22355444 10.3390/v3122374 PMC3280510

[30] Mathew A , West K , Kalayanarooj S *et al* B‐cell responses during primary and secondary dengue virus infections in humans. J Infect Dis 2011; 204: 1514–1522. 21930609 10.1093/infdis/jir607 PMC3222107

[31] Vaughan K , Greenbaum J , Blythe M , Peters B , Sette A . Meta‐analysis of all immune epitope data in the *Flavivirus* genus: inventory of current immune epitope data status in the context of virus immunity and immunopathology. Viral Immunol 2010; 23: 259–284. 20565291 10.1089/vim.2010.0006 PMC2942863

[32] Garcia‐Sastre A , Biron CA . Type 1 interferons and the virus‐host relationship: a lesson in detente. Science 2006; 312: 879–882. 16690858 10.1126/science.1125676

[33] Smith DR , Hollidge B , Daye S *et al* Neuropathogenesis of Zika virus in a highly susceptible immunocompetent mouse model after antibody blockade of type I interferon. PLoS Negl Trop Dis 2017; 11: e0005296. 28068342 10.1371/journal.pntd.0005296 PMC5249252

[34] Govero J , Esakky P , Scheaffer SM *et al* Zika virus infection damages the testes in mice. Nature 2016; 540: 438–442. 27798603 10.1038/nature20556 PMC5432198

[35] Hastings AK , Yockey LJ , Jagger BW *et al* TAM receptors are not required for Zika virus infection in mice. Cell Rep 2017; 19: 558–568. 28423319 10.1016/j.celrep.2017.03.058 PMC5485843

[36] Gorman MJ , Caine EA , Zaitsev K *et al* An immunocompetent mouse model of Zika virus infection. Cell Host Microbe 2018; 23: 672–685.e6. 29746837 10.1016/j.chom.2018.04.003 PMC5953559

[37] Scott JM , Lebratti TJ , Richner JM *et al* Cellular and humoral immunity protect against vaginal Zika virus infection in mice. J Virol 2018; 92: e00038‐18. 29343577 10.1128/JVI.00038-18 PMC5972878

[38] Long F , Doyle M , Fernandez E *et al* Structural basis of a potent human monoclonal antibody against Zika virus targeting a quaternary epitope. Proc Natl Acad Sci USA 2019; 116: 1591–1596. 30642974 10.1073/pnas.1815432116 PMC6358714

[39] Roehrig JT , Nash D , Maldin B *et al* Persistence of virus‐reactive serum immunoglobulin m antibody in confirmed west Nile virus encephalitis cases. Emerg Infect Dis 2003; 9: 376–379. 12643836 10.3201/eid0903.020531 PMC2958550

[40] Prince HE , Matud JL . Estimation of dengue virus IgM persistence using regression analysis. Clin Vaccine Immunol 2011; 18: 2183–2185. 22030368 10.1128/CVI.05425-11 PMC3232704

[41] Jego G , Pascual V , Palucka AK , Banchereau J . Dendritic cells control B cell growth and differentiation. Curr Dir Autoimmun 2005; 8: 124–139. 15564719 10.1159/000082101

[42] Welten SPM , Redeker A , Toes REM , Arens R . Viral persistence induces antibody inflation without altering antibody avidity. J Virol 2016; 90: 4402–4411. 26889035 10.1128/JVI.03177-15 PMC4836336

[43] Nish S , Medzhitov R . Host defense pathways: role of redundancy and compensation in infectious disease phenotypes. Immunity 2011; 34: 629–636. 21616433 10.1016/j.immuni.2011.05.009 PMC3143490

[44] Coutelier JP , van der Logt JT , Heessen FW , Warnier G , Van Snick J . IgG2a restriction of murine antibodies elicited by viral infections. J Exp Med 1987; 165: 64–69. 3794607 10.1084/jem.165.1.64 PMC2188250

[45] Klaus GG , Pepys MB , Kitajima K , Askonas BA . Activation of mouse complement by different classes of mouse antibody. Immunology 1979; 38: 687–695. 521057 PMC1457877

[46] Lum FM , Teo TH , Lee WW *et al* An essential role of antibodies in the control of Chikungunya virus infection. J Immunol 2013; 190: 6295–6302. 23670192 10.4049/jimmunol.1300304 PMC3677171

[47] Chen HW , Huang HW , Hu HM *et al* A poorly neutralizing IgG2a/c response elicited by a DNA vaccine protects mice against Japanese encephalitis virus. J Gen Virol 2014; 95: 1983–1990. 24914069 10.1099/vir.0.067280-0

[48] Swanson CL , Wilson TJ , Strauch P *et al* Type I IFN enhances follicular B cell contribution to the T cell‐independent antibody response. J Exp Med 2010; 207: 1485–1500. 20566717 10.1084/jem.20092695 PMC2901065

[49] Malkiel S , Diamond B . Chapter 24 ‐ Anti‐DNA antibodies In: Tsokos GC, editor. Systemic Lupus Erythematosus. Boston: Academic Press; 2016: 207–211.

[50] Beasley DW , Barrett AD . Identification of neutralizing epitopes within structural domain III of the West Nile virus envelope protein. J Virol 2002; 76: 13097–13100. 12438639 10.1128/JVI.76.24.13097-13100.2002 PMC136710

[51] Pierson TC , Diamond MS . Molecular mechanisms of antibody‐mediated neutralisation of flavivirus infection. Expert Rev Mol Med 2008; 10: e12. 18471342 10.1017/S1462399408000665 PMC2671962

[52] Imbach PA . Harmful and beneficial antibodies in immune thrombocytopenic purpura. Clin Exp Immunol 1994; 97(Suppl 1): 25–30. 8033430 PMC1550381

[53] Casadevall A , Janda A . Immunoglobulin isotype influences affinity and specificity. Proc Natl Acad Sci USA 2012; 109: 12272–12273. 22826242 10.1073/pnas.1209750109 PMC3412040

[54] Peng SL , Szabo SJ , Glimcher LH . T‐bet regulates IgG class switching and pathogenic autoantibody production. Proc Natl Acad Sci USA 2002; 99: 5545–5550. 11960012 10.1073/pnas.082114899 PMC122806

[55] Shinomiya N , Kuratsuji T , Yata J‐I . The role of T cells in immunoglobulin class switching of specific antibody production system *in vitro* in humans. Cell Immunol 1989; 118: 239–249. 2521307 10.1016/0008-8749(89)90375-4

[56] Taylor JM , Ziman ME , Canfield DR , Vajdy M , Solnick JV . Effects of a Th1‐ versus a Th2‐biased immune response in protection against Helicobacter pylori challenge in mice. Microb Pathog 2008; 44: 20–27. 17683897 10.1016/j.micpath.2007.06.006 PMC2234601

[57] Jeurissen A , Ceuppens JL , Bossuyt X . T lymphocyte dependence of the antibody response to ‘T lymphocyte independent type 2’ antigens. Immunology 2004; 111: 1–7. 14678191 10.1111/j.1365-2567.2003.01775.x PMC1782396

[58] Mesin L , Ersching J , Victora GD . Germinal center B cell dynamics. Immunity 2016; 45: 471–482. 27653600 10.1016/j.immuni.2016.09.001 PMC5123673

[59] Elong Ngono A , Young MP , Bunz M *et al* CD4^+^ T cells promote humoral immunity and viral control during Zika virus infection. PLoS Pathog 2019; 15: e1007474. 30677097 10.1371/journal.ppat.1007474 PMC6345435

[60] Chung KM , Thompson BS , Fremont DH , Diamond MS . Antibody recognition of cell surface‐associated NS1 triggers Fc‐γ receptor‐mediated phagocytosis and clearance of West Nile Virus‐infected cells. J Virol 2007; 81: 9551–9555. 17582005 10.1128/JVI.00879-07 PMC1951387

[61] Rodriguez‐Madoz JR , Belicha‐Villanueva A , Bernal‐Rubio D *et al* Inhibition of the type I interferon response in human dendritic cells by dengue virus infection requires a catalytically active NS2B3 complex. J Virol 2010; 84: 9760–9774. 20660196 10.1128/JVI.01051-10 PMC2937777

[62] Castillo Ramirez JA , Urcuqui‐Inchima S . Dengue virus control of type I IFN responses: a history of manipulation and control. J Interferon Cytokine Res 2015; 35: 421–430. 25629430 10.1089/jir.2014.0129 PMC4490770

[63] de Alwis R , Beltramello M , Messer WB *et al* In‐depth analysis of the antibody response of individuals exposed to primary dengue virus infection. PLoS Negl Trop Dis 2011; 5: e1188. 21713020 10.1371/journal.pntd.0001188 PMC3119640

[64] Yuki N . Guillain‐Barré syndrome and anti‐ganglioside antibodies: a clinician‐scientist's journey. Proc Jpn Acad Ser B Phys Biol Sci 2012; 88: 299–326. 10.2183/pjab.88.299 PMC3422685 22850724

[65] Kiefer K , Oropallo MA , Cancro MP , Marshak‐Rothstein A . Role of type I interferons in the activation of autoreactive B cells. Immunol Cell Biol 2012; 90: 498–504. 22430248 10.1038/icb.2012.10 PMC3701256

[66] Tan JJL , Balne PK , Leo Y‐S *et al* Persistence of Zika virus in conjunctival fluid of convalescence patients. Sci Rep 2017; 7: 11194. 28894118 10.1038/s41598-017-09479-5 PMC5594005

[67] Martin RM , Brady JL , Lew AM . The need for IgG2c specific antiserum when isotyping antibodies from C57BL/6 and NOD mice. J Immunol Methods 1998; 212: 187–192. 9672206 10.1016/s0022-1759(98)00015-5

[68] Lum FM , Lin C , Susova OY *et al* A sensitive method for detecting Zika virus antigen in patients' whole‐blood specimens as an alternative diagnostic approach. J Infect Dis 2017; 216: 182–190. 28586426 10.1093/infdis/jix276 PMC5853302

