# Supplementary material for: Type I interferon shapes the quantity and quality of the anti‐Zika virus antibody response
Source: Clin Transl Immunology. 2020 Apr 26;9(4):e1126. doi: 10.1002/cti2.1126 (PMC7184064; doi:10.1002/cti2.1126)
Supplement: Supplementary file 1 — Fig S1 [file CTI2-9-e1126-s001.pptx]

## Slide 1
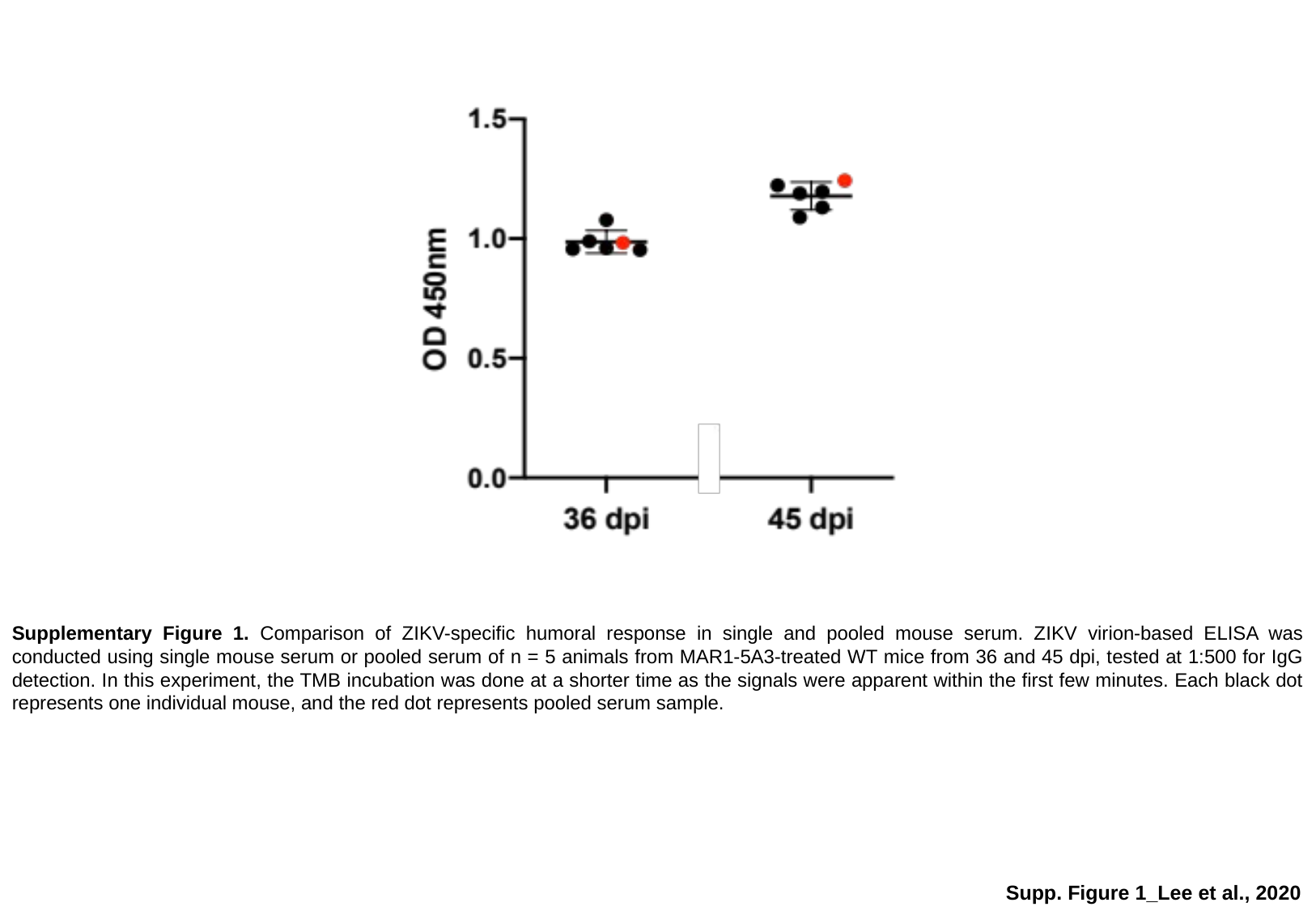

Supplementary Figure 1. Comparison of ZIKV-specific humoral response in single and pooled mouse serum. ZIKV virion-based ELISA was conducted using single mouse serum or pooled serum of n = 5 animals from MAR1-5A3-treated WT mice from 36 and 45 dpi, tested at 1:500 for IgG detection. In this experiment, the TMB incubation was done at a shorter time as the signals were apparent within the first few minutes. Each black dot represents one individual mouse, and the red dot represents pooled serum sample.
Supp. Figure 1_Lee et al., 2020
