# Supplementary material for: Type I interferon shapes the quantity and quality of the anti‐Zika virus antibody response
Source: Clin Transl Immunology. 2020 Apr 26;9(4):e1126. doi: 10.1002/cti2.1126 (PMC7184064; doi:10.1002/cti2.1126)
Supplement: Supplementary file 2 — Fig S2 [file CTI2-9-e1126-s002.pptx]

## Slide 1
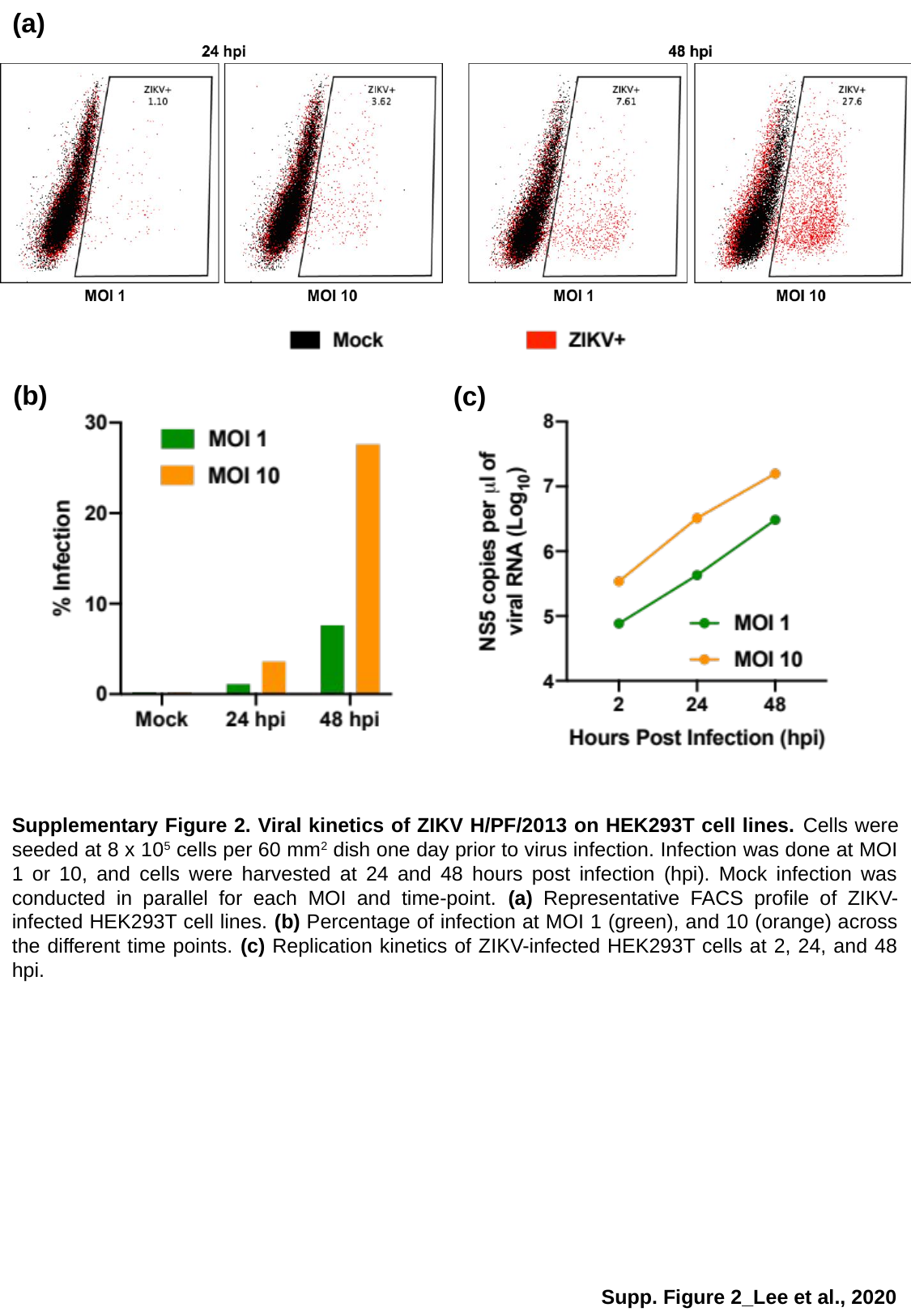

(a)
(b)
(c)
Supplementary Figure 2. Viral kinetics of ZIKV H/PF/2013 on HEK293T cell lines. Cells were seeded at 8 x 10^5^ cells per 60 mm2 dish one day prior to virus infection. Infection was done at MOI 1 or 10, and cells were harvested at 24 and 48 hours post infection (hpi). Mock infection was conducted in parallel for each MOI and time-point. (a) Representative FACS profile of ZIKV-infected HEK293T cell lines. (b) Percentage of infection at MOI 1 (green), and 10 (orange) across the different time points. (c) Replication kinetics of ZIKV-infected HEK293T cells at 2, 24, and 48 hpi.
Supp. Figure 2_Lee et al., 2020
