# Supplementary material for: Type I interferon shapes the quantity and quality of the anti‐Zika virus antibody response
Source: Clin Transl Immunology. 2020 Apr 26;9(4):e1126. doi: 10.1002/cti2.1126 (PMC7184064; doi:10.1002/cti2.1126)
Supplement: Supplementary file 3 — Fig S3 [file CTI2-9-e1126-s003.pptx]

## Slide 1
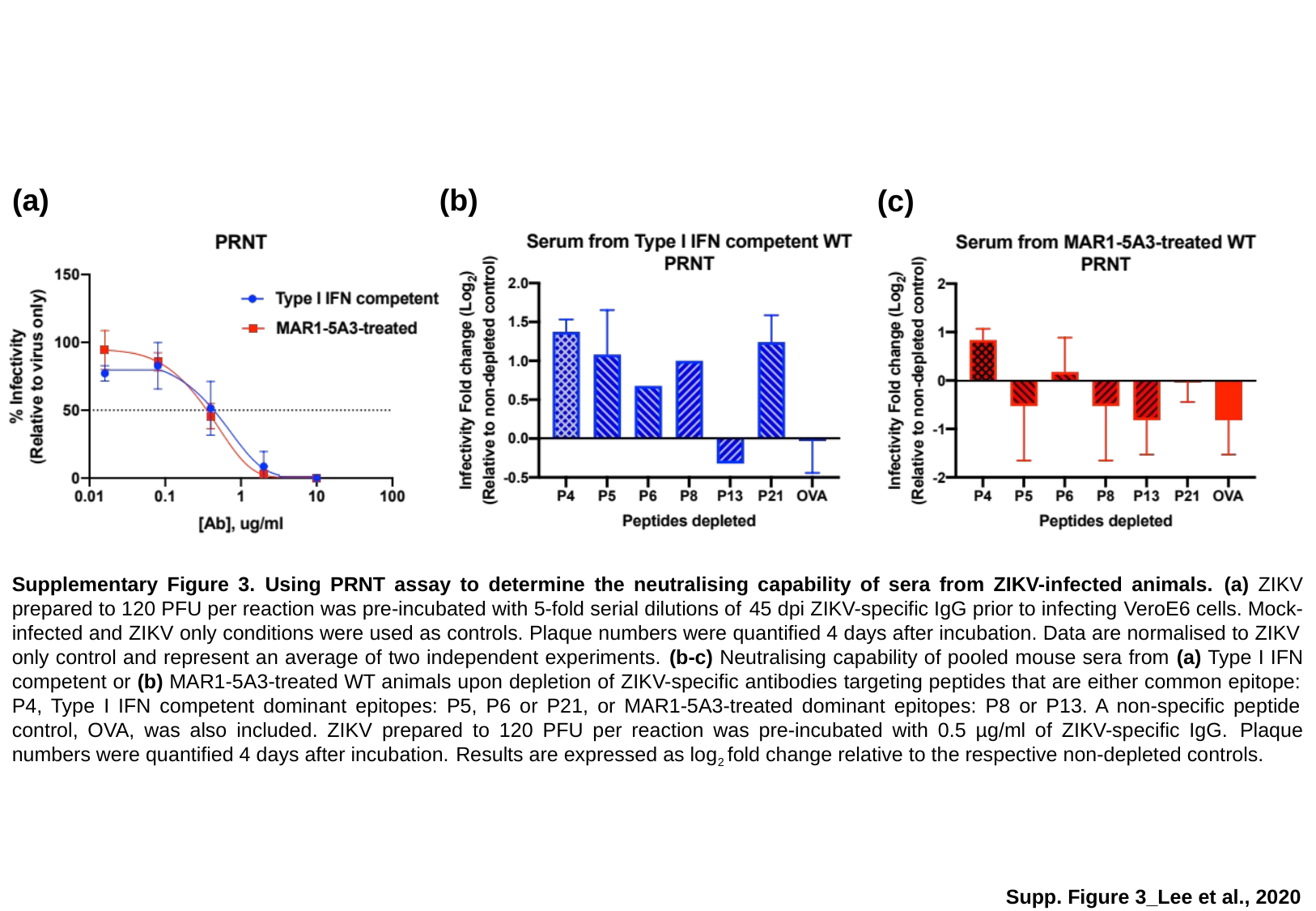

(a)
(b)
(c)
Supplementary Figure 3. Using PRNT assay to determine the neutralising capability of sera from ZIKV-infected animals. (a) ZIKV prepared to 120 PFU per reaction was pre-incubated with 5-fold serial dilutions of 45 dpi ZIKV-specific IgG prior to infecting VeroE6 cells. Mock-infected and ZIKV only conditions were used as controls. Plaque numbers were quantified 4 days after incubation. Data are normalised to ZIKV only control and represent an average of two independent experiments. (b-c) Neutralising capability of pooled mouse sera from (a) Type I IFN competent or (b) MAR1-5A3-treated WT animals upon depletion of ZIKV-specific antibodies targeting peptides that are either common epitope: P4, Type I IFN competent dominant epitopes: P5, P6 or P21, or MAR1-5A3-treated dominant epitopes: P8 or P13. A non-specific peptide control, OVA, was also included. ZIKV prepared to 120 PFU per reaction was pre-incubated with 0.5 µg/ml of ZIKV-specific IgG. Plaque numbers were quantified 4 days after incubation. Results are expressed as log2 fold change relative to the respective non-depleted controls.
Supp. Figure 3_Lee et al., 2020
