# Supplementary material for: Type I interferon shapes the quantity and quality of the anti‐Zika virus antibody response
Source: Clin Transl Immunology. 2020 Apr 26;9(4):e1126. doi: 10.1002/cti2.1126 (PMC7184064; doi:10.1002/cti2.1126)
Supplement: Supplementary file 4 — Fig S4 [file CTI2-9-e1126-s004.pptx]

## Slide 1
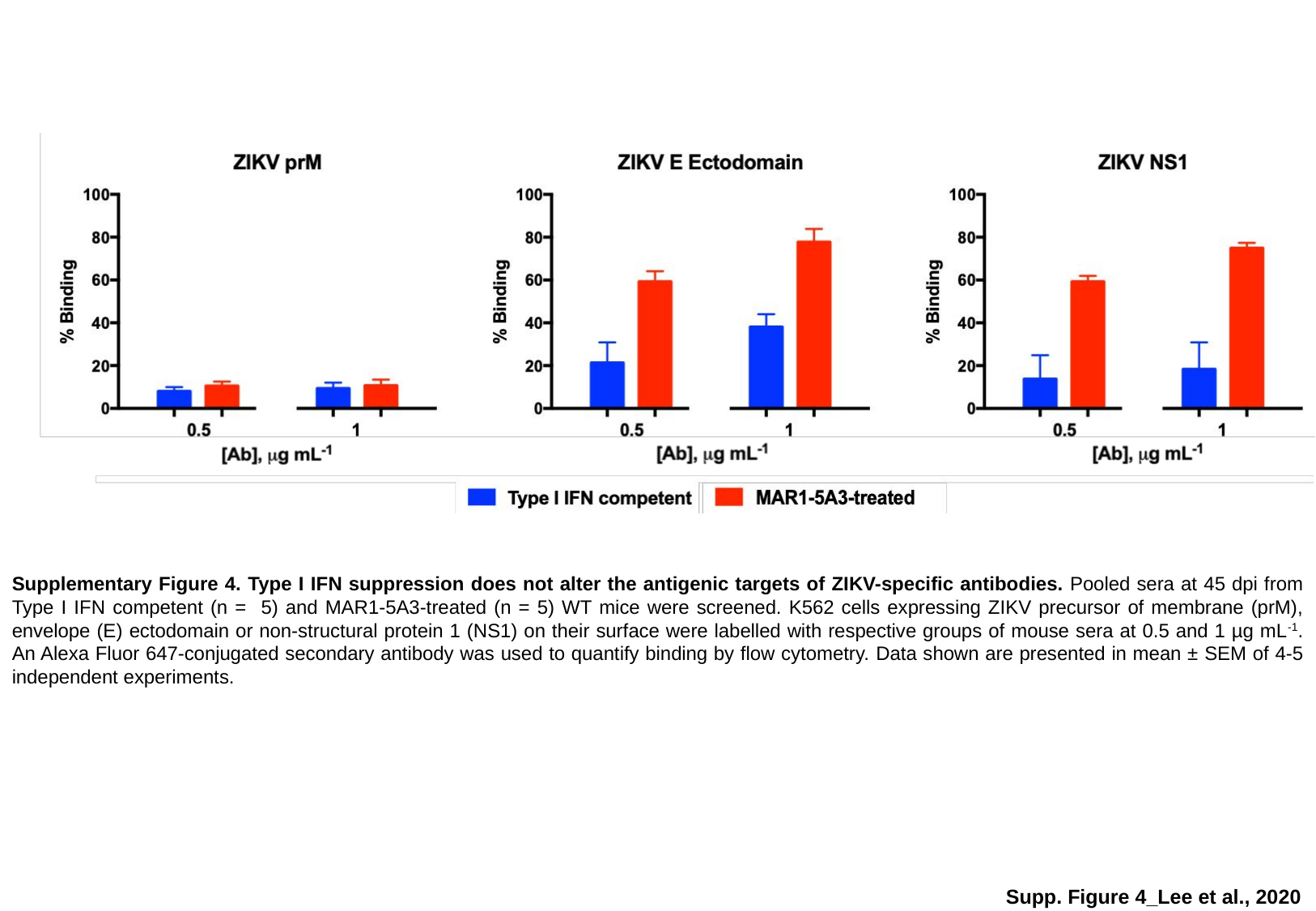

Supplementary Figure 4. Type I IFN suppression does not alter the antigenic targets of ZIKV-specific antibodies. Pooled sera at 45 dpi from Type I IFN competent (n = 5) and MAR1-5A3-treated (n = 5) WT mice were screened. K562 cells expressing ZIKV precursor of membrane (prM), envelope (E) ectodomain or non-structural protein 1 (NS1) on their surface were labelled with respective groups of mouse sera at 0.5 and 1 µg mL-1. An Alexa Fluor 647-conjugated secondary antibody was used to quantify binding by flow cytometry. Data shown are presented in mean ± SEM of 4-5 independent experiments.
Supp. Figure 4_Lee et al., 2020
