# Supplementary material for: Type I interferon shapes the quantity and quality of the anti‐Zika virus antibody response
Source: Clin Transl Immunology. 2020 Apr 26;9(4):e1126. doi: 10.1002/cti2.1126 (PMC7184064; doi:10.1002/cti2.1126)
Supplement: Supplementary file 5 — Fig S5 [file CTI2-9-e1126-s005.pptx]

## Slide 1
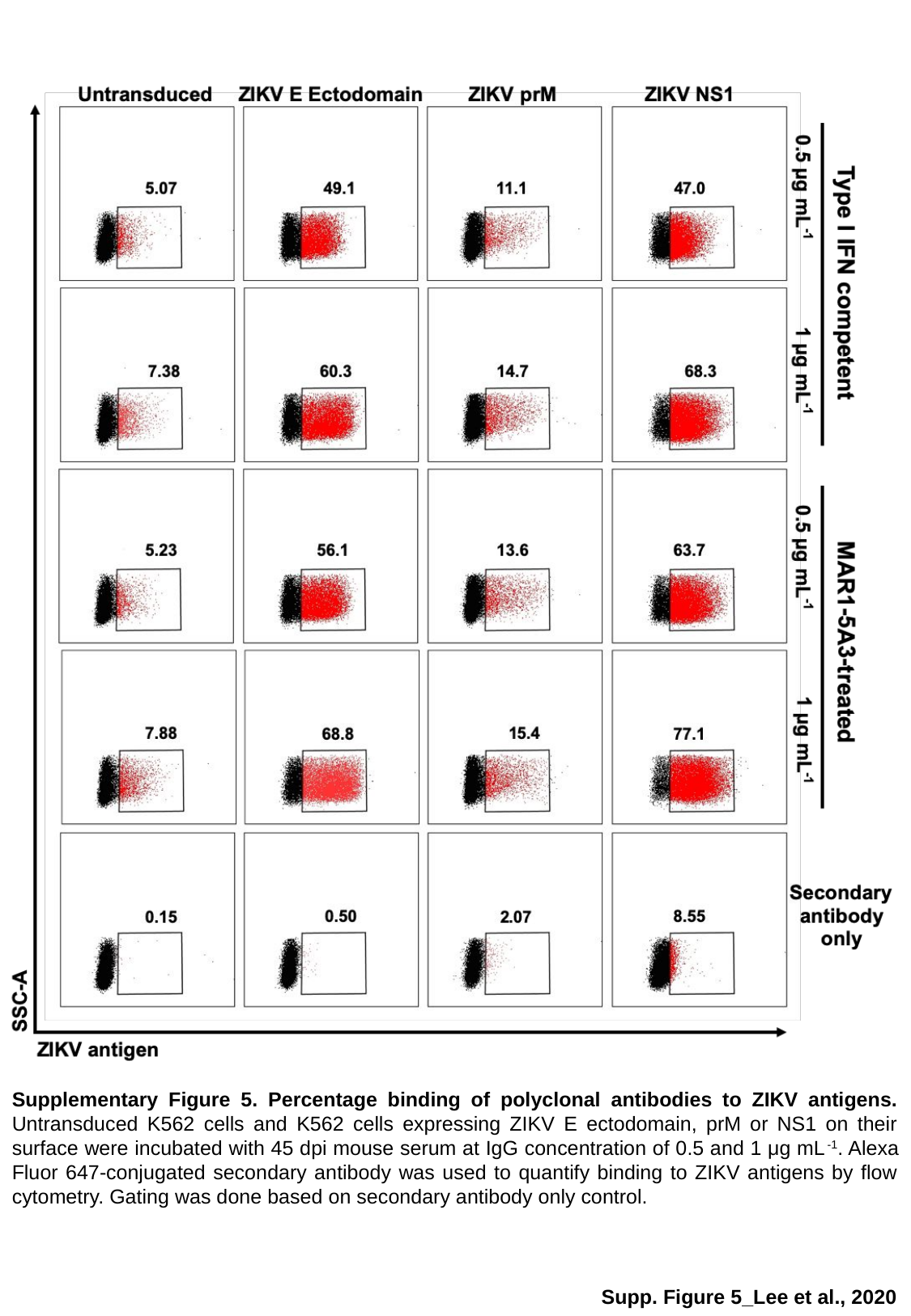

Supplementary Figure 5. Percentage binding of polyclonal antibodies to ZIKV antigens. Untransduced K562 cells and K562 cells expressing ZIKV E ectodomain, prM or NS1 on their surface were incubated with 45 dpi mouse serum at IgG concentration of 0.5 and 1 μg mL-1. Alexa Fluor 647-conjugated secondary antibody was used to quantify binding to ZIKV antigens by flow cytometry. Gating was done based on secondary antibody only control.
Supp. Figure 5_Lee et al., 2020
