# Supplementary material for: Type I interferon shapes the quantity and quality of the anti‐Zika virus antibody response
Source: Clin Transl Immunology. 2020 Apr 26;9(4):e1126. doi: 10.1002/cti2.1126 (PMC7184064; doi:10.1002/cti2.1126)
Supplement: Supplementary file 6 — Fig S6 [file CTI2-9-e1126-s006.pptx]

## Slide 1
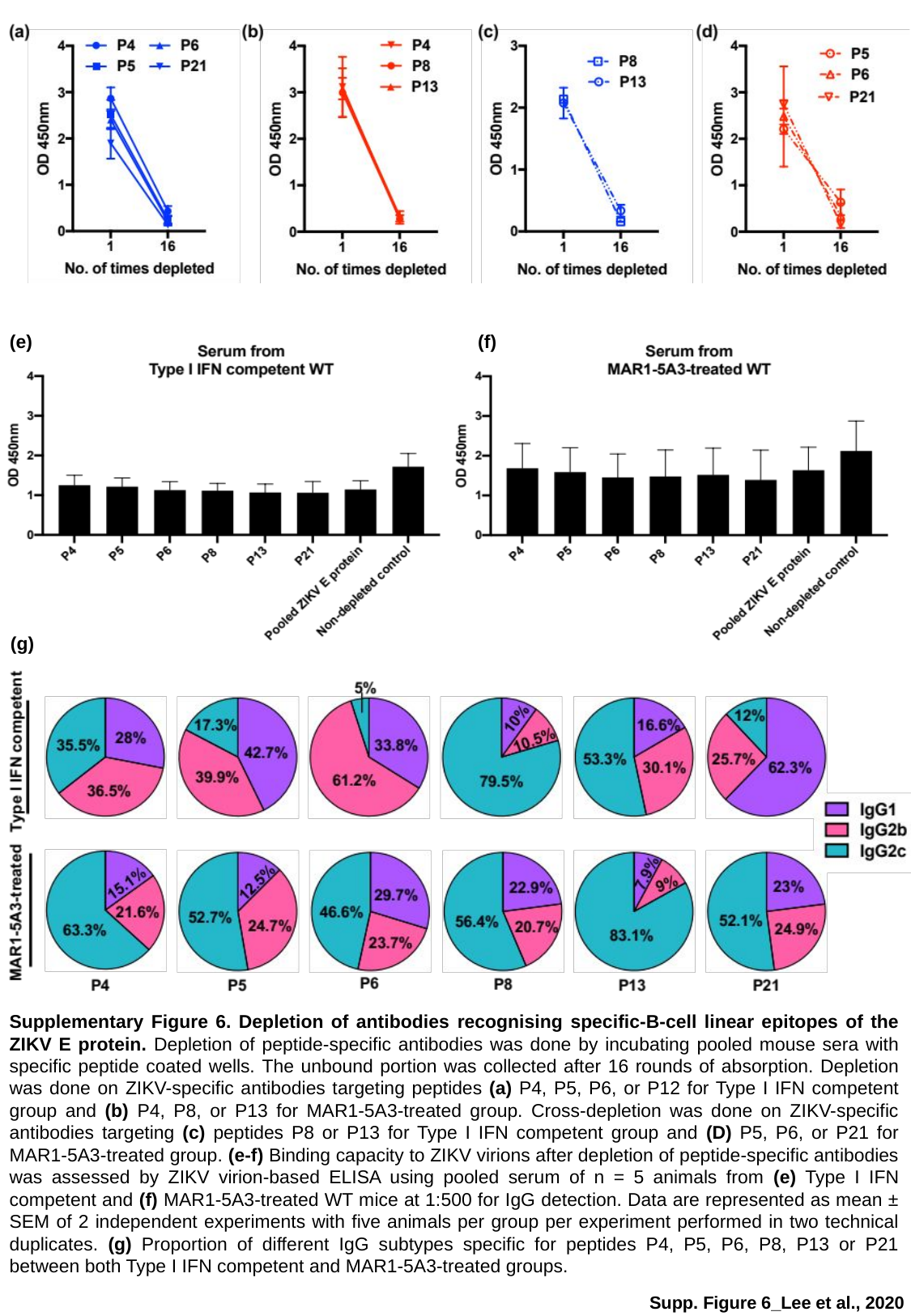

(f)
(e)
(g)
Supplementary Figure 6. Depletion of antibodies recognising specific-B-cell linear epitopes of the ZIKV E protein. Depletion of peptide-specific antibodies was done by incubating pooled mouse sera with specific peptide coated wells. The unbound portion was collected after 16 rounds of absorption. Depletion was done on ZIKV-specific antibodies targeting peptides (a) P4, P5, P6, or P12 for Type I IFN competent group and (b) P4, P8, or P13 for MAR1-5A3-treated group. Cross-depletion was done on ZIKV-specific antibodies targeting (c) peptides P8 or P13 for Type I IFN competent group and (D) P5, P6, or P21 for MAR1-5A3-treated group. (e-f) Binding capacity to ZIKV virions after depletion of peptide-specific antibodies was assessed by ZIKV virion-based ELISA using pooled serum of n = 5 animals from (e) Type I IFN competent and (f) MAR1-5A3-treated WT mice at 1:500 for IgG detection. Data are represented as mean ± SEM of 2 independent experiments with five animals per group per experiment performed in two technical duplicates. (g) Proportion of different IgG subtypes specific for peptides P4, P5, P6, P8, P13 or P21 between both Type I IFN competent and MAR1-5A3-treated groups.
Supp. Figure 6_Lee et al., 2020
