# Supplementary material for: Type I interferon shapes the quantity and quality of the anti‐Zika virus antibody response
Source: Clin Transl Immunology. 2020 Apr 26;9(4):e1126. doi: 10.1002/cti2.1126 (PMC7184064; doi:10.1002/cti2.1126)
Supplement: Supplementary file 7 — Table S1 [file CTI2-9-e1126-s007.docx]

**Supplementary Table 1. Amount of antibodies pre- and post-depletion of ZIKV-specific antibodies**

|  | **Batch 1 Type I IFN competent** | **Batch 1 Type I IFN competent; Depleted** | **Batch 2 Type I IFN competent** | **Batch 2 Type I IFN competent; Depleted** | **Batch 3 Type I IFN competent** | **Batch 3 Type I IFN competent; Depleted** |
| --- | --- | --- | --- | --- | --- | --- |
| **Interpolated** | -1.31877299 | -1.35943386 | -1.15947867 | -1.36970798 | -1.27435553 | -1.33330622 |
| **Anti-log** | 0.048 | 0.04371 | 0.06927 | 0.04269 | 0.05317 | 0.04642 |
| **x10,000** | 480 | 437.1 | 692.7 | 426.9 | 531.7 | 464.2 |
|  |  |  |  |  |  |  |
|  | **Batch 1 MAR1-5A3-treated** | **Batch 1 MAR1-5A3-treated; Depleted** | **Batch 2 MAR1-5A3-treated** | **Batch 2 MAR1-5A3-treated; Depleted** | **Batch 3 MAR1-5A3-treated** | **Batch 3 MAR1-5A3-treated; Depleted** |
| **Interpolated** | -0.96104359 | -1.3456909 | -0.88563169 | -1.12786542 | -0.9173668 | -1.25768205 |
| **Anti-log** | 0.10938 | 0.04511 | 0.13013 | 0.0745 | 0.12096 | 0.05525 |
| **x10,000** | 1093.8 | 451.1 | 1301.3 | 745 | 1209.6 | 552.5 |
|  |  |  |  |  |  |  |
|  | **Batch 1 Type I IFN competent** | **Batch 2 Type I IFN competent** | **Batch 3 Type I IFN competent** | **Batch 1 MAR1-5A3-treated** | **Batch 2 MAR1-5A3-treated** | **Batch 3 MAR1-5A3-treated** |
| **ZIKV specific IgG (μg/ml)** | 42.9 | 265.8 | 67.5 | 642.7 | 556.3 | 657.1 |
