# Supplementary material for: Type I interferon shapes the quantity and quality of the anti‐Zika virus antibody response
Source: Clin Transl Immunology. 2020 Apr 26;9(4):e1126. doi: 10.1002/cti2.1126 (PMC7184064; doi:10.1002/cti2.1126)
Supplement: Supplementary file 8 — Table S2 [file CTI2-9-e1126-s008.docx]

**Supplementary Table 2. Annotation of individual peptides for mapping of ZIKV B-cell linear epitopes on the ZIKV E and NS1 proteome**

| **Protein** | **Peptide No.** | **Amino acid position on ZIKV (accession: KJ776791)** | | **Polypeptide sequence*^a^*** |
| --- | --- | --- | --- | --- |
|  |  | **Start** | **End** |  |
| **E** | P1 | 37 | 54 | DKPTVDIELVTTTVSNMA |
|  | P2 | 55 | 72 | EVRSYCYEASISDMASDS |
|  | P3 | 73 | 90 | RCPTQGEAYLDKQSDTQY |
|  | P4 | 91 | 108 | VCKRTLVDRGWGNGCGLF |
|  | P5 | 113 | 130 | LVTCAKFACSKKMTGKSI |
|  | P6 | 123 | 140 | KKMTGKSIQPENLEYRIM |
|  | P7 | 131 | 149 | PENLEYRIMLSVHGSQHS |
|  | P8 | 149 | 166 | SGMIVNDTGHETDENRAK |
|  | P9 | 157 | 174 | GHETDENRAKVEITPNSP |
|  | P10 | 166 | 183 | KVEITPNSPRAEATLGGF |
|  | P11 | 191 | 203 | EPRTGLDFSDLYY |
|  | P12 | 199 | 216 | SDLYYLTMNNKHWLVHKE |
|  | P13 | 217 | 234 | WFHDIPLPWHAGADTGTP |
|  | P14 | 235 | 245 | HWNNKEALVEF |
|  | P15 | 244 | 261 | EFKDAHAKRQTVVVLGSQ |
|  | P16 | 271 | 288 | GALEAEMDGAKGRLSSGH |
|  | P17 | 306 | 319 | SLCTAAFTFTKIPA |
|  | P18 | 325 | 342 | TVTVEVQYAGTDGPCKVP |
|  | P19 | 343 | 355 | AQMAVDMQTLTPV |
|  | P20 | 361 | 378 | ANPVITESTENSKMMLEL |
|  | P21 | 402 | 419 | RSGSTIGKAFEATVRGAK |
|  | P22 | 453 | 470 | FKSLFGGMSWFSQILIGT |
| **NS1** | P23 | 1 | 18 | DVGCSVDFSKKETRCGTG |
|  | P24 | 19 | 36 | VFVYNDVEAWRDRYKYHP |
|  | P25 | 55 | 72 | CGISSVSRMENIMWRSVE |
|  | P26 | 73 | 90 | GELNAILEENGVQLTVVV |
|  | P27 | 91 | 112 | GSVKNPMWRGPQRLPVPVNELP |
|  | P28 | 119 | 136 | GKSYFVRAAKTNNSFVVD |
|  | P29 | 137 | 154 | GDTLKECPLKHRAWNSFL |
|  | P30 | 155 | 176 | VEDHGFGVFHTSVWLKVREDYS |
|  | P31 | 239 | 256 | SDLIIPKSLAGPLSHHNT |
|  | P32 | 248 | 265 | AGPLSHHNTREGYRTQMK |
|  | P33 | 257 | 274 | REGYRTQMKGPWHSEELE |
|  | P34 | 266 | 283 | GPWHSEELEIRFEECPGT |
|  | P35 | 275 | 292 | IRFEECPGTKVHVEETCG |
|  | P36 | 284 | 301 | KVHVEETCGTRGPSLRST |
|  | P37 | 293 | 310 | TRGPSLRSTTASGRVIEE |
|  | P38 | 302 | 319 | TASGRVIEEWCCRECTMP |
|  | P39 | 311 | 328 | WCCRECTMPPLSFRAKDG |
|  | P40 | 320 | 337 | PLSFRAKDGCWYGMEIRP |
|  | P41 | 329 | 346 | CWYGMEIRPRKEPESNLV |

*^a^* The numbers correspond to the amino acid positions along the ZIKV viral genome.
