# Supplementary material for: Type I interferon shapes the quantity and quality of the anti‐Zika virus antibody response
Source: Clin Transl Immunology. 2020 Apr 26;9(4):e1126. doi: 10.1002/cti2.1126 (PMC7184064; doi:10.1002/cti2.1126)
Supplement: Supplementary file 9 — Table S3 [file CTI2-9-e1126-s009.docx]

**Supplementary Table 3. Differential recognition levels against B-cell linear epitopes on the ZIKV NS1 protein between Type I IFN competent and MAR1-5A3-treated WT mice sera**

| **Type I IFN competent (%)** | **Peptide** | **MAR1-5A3-treated (%)** | **Peptide** | **Recognition level** |
| --- | --- | --- | --- | --- |
| 43.39150983 | **P24** | 17.97125222 | **P37** | High (> 10%) |
| 22.94882271 | **P27** | 14.65112428 | **P24** |  |
| 17.95603248 | **P36** | 12.30728132 | **P28** |  |
| 17.03081138 | **P26** |  |  |  |
| 11.75628739 | **P30** |  |  |  |
| 8.810444034 | **P32** | 8.911950314 | **P30** | Moderate (> 5%) |
| 7.449143609 | **P33** |  |  |  |
| 6.371314740 | **P40** |  |  |  |
| 6.242329041 | **P37** |  |  |  |
| 4.019662904 | **P31** | 4.981932005 | **P38** | Low (> 1%) |
| 3.849885547 | **P41** | 3.931547081 | **P32** |  |
| 3.668491126 | **P28** | 3.842258950 | **P39** |  |
| 3.473736957 | **P34** | 3.668641605 | **P36** |  |
| 3.182860648 | **P38** | 3.645884664 | **P29** |  |
|  |  | 3.443826605 | **P26** |  |
|  |  | 2.956295673 | **P35** |  |
|  |  | 2.910509751 | **P40** |  |
|  |  | 2.907565022 | **P34** |  |
|  |  | 2.772167552 | **P41** |  |
|  |  | 2.736408032 | **P27** |  |
|  |  | 2.407933033 | **P25** |  |
|  |  | 2.384982530 | **P33** |  |
|  |  | 2.247592500 | **P23** |  |
|  |  | 1.758005269 | **P31** |  |
| 0.883817161 | **P39** |  |  | No binding (< 1%) |
| 0.768270281 | **P29** |  |  |  |
| 0.022375118 | **P23** |  |  |  |
| 0 | **P25** |  |  |  |
| 0 | **P35** |  |  |  |
